# Highly fractionated chromium isotopes in Mesoproterozoic-aged shales and atmospheric oxygen

**DOI:** 10.1038/s41467-018-05263-9

**Published:** 2018-07-20

**Authors:** Donald E. Canfield, Shuichang Zhang, Anja B. Frank, Xiaomei Wang, Huajian Wang, Jin Su, Yuntao Ye, Robert Frei

**Affiliations:** 1 Key Laboratory of Petroleum Geochemistry, Research Institute of Petroleum Exploration and Development, China National Petroleum Corporation, 100083 Beijing, China; 2 Nordcee, Institute of Biology, University of Southern Denmark, Campusvej 55, 5230 Odense M, Denmark; 3 Department of Geoscience and Natural Resource Management, University of Copenhagen, Øster Voldgade 10, 1350 Copenhagen K, Denmark

## Abstract

The history of atmospheric oxygen through the Mesoproterozoic Era is uncertain, but may have played a role in the timing of major evolutionary developments among eukaryotes. Previous work using chromium isotopes in sedimentary rocks has suggested that Mesoproterozoic Era atmospheric oxygen levels were too  low in concentration (<0.1% of present-day levels (PAL)) for the expansion of eukaryotic algae and for the evolution of crown-group animals that occurred later in the Neoproterozoic Era. In contrast, our new results on chromium isotopes from Mesoproterozoic-aged sedimentary rocks from the Shennongjia Group from South China is consistent with atmospheric oxygen concentrations of >1% PAL and thus the possibility that a permissive environment existed long before the expansion of various eukaryotic clades.

## Abstract (web summary)

There is a long standing debate whether low atmospheric oxygen levels during the Mesoproterozoic Era hindered the evolution of crown-group animals. Here, the authors show with shale-hosted chromium isotopes that sufficient atmospheric oxygen for crown-group animals likely predated their evolution by over 400 million years.

## Introduction

Atmospheric oxygen has played a key role in structuring the ecology and biogeochemical functioning of marine ecosystems through time. For example, expanded ocean anoxia under lower atmospheric oxygen concentrations^1^ could reduce nitrogen availability through higher rates of fixed nitrogen conversion to N_2_ by denitrification and anammox^2^. In addition, enhanced phosphorus removal by adsorption onto Fe oxides under expanded ocean anoxia could reduce phosphorus availability^3,4^. Either singly, or in combination, nitrogen and/or phosphorus limitation could have impacted rates of primary production in the oceans under reduced oxygen levels^2–4^.

Oxygen limitation might also have impacted the pace and timing of eukaryote evolution^5^, with a long-standing view that critical stages of animal evolution were enabled by increases in atmospheric oxygen during the Neoproterozoic Era^5–8^. This idea was initially conceived under the assumption that atmospheric oxygen only first accumulated during the Neoproterozoic Era, allowing only then the evolution of organisms with aerobic respiration including animals^6^.

A more modern view recognizes that atmospheric oxygen first began accumulating some 2400 Ma^9^, and that various stages of animal evolution likely had different oxygen requirements^10,11^. Thus, early stem-group animals may have lacked complex multicellularity^12,13^ and may have had oxygen requirements closer to their single-celled choanoflagellate sister group. Later stem-group animals, and crown-group animals, with their complex multicellularity^14^, likely had higher oxygen demands^15^, while large motile animals with high exercise metabolism likely had higher oxygen requirements still^14,16,17^. Molecular clock estimates place the origin of stem-group animals at around 900 Ma^18^, the origin of crown-group animals at around 750–800 Ma^16,18^, while the early Cambrian Period (541–485.5 Ma) represents the evolution of widespread animal motility^16^.

Thus, oxygen availability could have potentially generated a barrier to animal evolution during several stages, ranging from the initial development of complex multicellularity in stem-group animals, to the attainment of motility, and even to episodes of animal gigantism occurring later in animal evolution^17,19^. If oxygen availability generated a barrier to any stage of animal evolution, one would expect a relationship between increases in oxygen concentration, lifting the barrier, and a particular stage in animal evolution and development. Exploring for such a relationship requires that we know the oxygen requirements for the various stages of early animal evolution and the history of atmospheric oxygen concentrations.

The oxygen requirements for early stem-group animals are not certain as the oxygen requirements of choanoflagellates have not been explored, but, with a typical choanoflagellate diameter of 5–10 μm^20^, an oxygen requirement of around 0.1–0.2% atmospheric levels (PAL) might be expected^21^. Likewise, the oxygen requirements of early crown-group animals are not known, but 0.36% PAL was calculated as the requirement of a small bilaterian animal (25 μm wide and 600 μm long) with a diffusional oxygen supply^22^. Through experimental studies, the minimal oxygen requirements for the sponge *Halochondria panacea* is <2–4% (PAL)^23^, and perhaps as low as 0.25% PAL for the sponge *Tethya wilhelma*^13^. To have played a role in early animal evolution, oxygen, then, should have been around 0.1–0.2% PAL (and sufficient for single-celled protists) before the development of complex multicellularity in stem-group animals rising to values of >0.4% PAL by the time crown-group animals diverged. Further rises, then, could have paced the attainment of various stages of animal motility and size.

The question is whether the history of atmospheric oxygen concentrations supports a relationship to early animal evolution. The history of chromium isotopes in various marine sediments presents an intriguing way to probe the history of atmospheric oxygenation. The basic premise is that the oxidative weathering of Cr(III) phases on land to Cr(VI) (chromate) generates an isotope fractionation such that the Cr(VI) is enriched in the heavy isotope ^53^Cr, leaving the residual Cr(III) ^53^Cr depleted. In the modern world, river waters are typically ^53^Cr-enriched^24^, as are the oceans^25,26^, while weathered soils may be ^53^Cr depleted^24,27^. Therefore, the accumulation of ^53^Cr-enriched authigenic chromium in marine sediments is a sign of the oxidative weathering of chromium on land^28^. The oxidative weathering of Cr(III) is, however, only indirectly related to atmospheric oxygen, where the proximal oxidant is MnO_2_^29^, but the rejuvenation of MnO_2_ requires oxygen. Other processes can also potentially influence chromium isotope systematics, and these will be discussed below.

The history of chromium isotopes as captured in ironstones and shales has revealed a general lack of fractionated chromium through the Mesoproterozoic Era (1600–1000 Ma), with the first indications of large fractionations around 750 Ma in the Neoproterozoic Era (1000–541 Ma)^8,30^. This absence of fractionated chromium in Mesoproterozoic-aged rocks implies no or limited oxidative weathering of Cr(III) on land and atmospheric oxygen levels of <0.1% PAL based on the oxygen requirements to rejuvenate MnO_2_ oxides and thus promote the oxidative weathering of Cr(III)^8^ (see below and Supplementary Discussion, Oxygen Concentration Model for further details). Oxygen levels this low would have likely prevented the emergence of crown-group animals as explored above (and possibly also protists in the 5–10 μm range). Furthermore, an increase in chromium isotope fractionation around 750 Ma^8,28,30^ signals a rise in atmospheric oxygen concentrations to levels sufficient to allow the emergence of crown animal groups, supporting a relationship between the two.

Subsequently, data from marine carbonates show highly ^53^Cr-enriched chromium in samples from several formations ranging in age from 1112 to 970 Ma^31^, suggesting higher oxygen levels at the Mesoproterozoic–Neoproterozoic boundary than indicated from previous Cr isotope studies. This study was careful to evaluate possible sources of late diagenetic ^53^Cr enrichments, but one might still argue that carbonate-hosted chromium could suffer from late diagenetic effects. We report here isotopically enriched chromium in Mesoproterozoic-aged shales dating back to 1350 Ma. These shales are from the Shennongjia Group of South China, and document elevated atmospheric oxygen levels through most of Mesoproterozoic Era; levels likely sufficient for early crown-group animal respiration, but attained long before they evolved.

## Results

### Study location

**Fig. 1:**
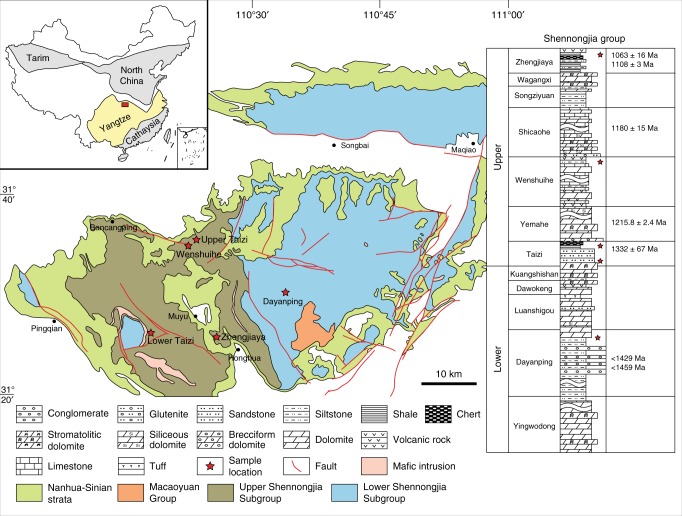
Map of Shennongjia Group including major tectonic features and a general stratigraphic column with dating. Indicated also on the figure are sampling locations as well as an indication as to where in the stratigraphy the samples were taken. The figure is modified from ref.^32^ with permission

We explored rocks from the Shennongjia Group (SG) of South China. The SG represents a series of Mesoproterozoic-aged sedimentary rocks from the Panxi-Hannan Belt in the northern margin of the Yangtze Block of the South China Craton (Fig. 1)^32^. The SG is housed in a structural dome of some 1800 km^2^ and is about 12,000-m thick. It is well exposed in the Shennongjia National Forest in the mountains of the western Hubei province, China (Fig. 1). The SG contains 11 formations and is informally divided into an upper section and a lower section (Fig. 1), separated by an uplift-generated disconformity surface^32^. Overall, the SG represents a series marine platform-margin deposits, ranging from shallow water to deep water, and whose sedimentology is described in reference^32^. We supplement these descriptions with our own field observations below.

With respect to chronology, the uppermost Zhengjiaya Formation (Fig. 1) houses andesitic pyroclastic rocks and metabasalts, whose zircons were dated to 1103 ± 8 Ma^33^ and 1063 ± 16 Ma^34^, respectively, using laser ablation inductively coupled plasma mass spectrometry (LA-ICP-MS). The Shicaohe Formation has a microprobe U-Pb date of 1180 ± 15 Ma on zircons extracted from a volcanic tuff^35^. This formation was intruded by mafic dikes with a zircon U-Pb age of 1083 ± 4.6 Ma, in one case, and a Baddeleyite U-Pb age of 1115 ± 9 Ma in another^36^. The Yemahe Formation has been dated to 1215.8 ± 2.4 Ma for zircons extracted from volcanic tuffs^36^, while a whole-rock U-Pb date on uranium-rich shales from the upper Taizi Formation yielded an age of 1332 ± 67 Ma^32^. Detrital zircons from the Daynping Formation in the lower section of the SG show a distribution of four peaks in age, the youngest of which, from two samples, are 1429 and 1459 Ma^36^, providing maximum age estimates for this formation.

It is suggested that sediments of the SG deposited on a microcontinent, and that during its final stages of deposition, the microcontinent assembled as part the South China Craton (and as part of the super-continent Rodinia) during the last stages of late Mesoproterozoic Grenville Orogeny^33,34^. This history is supported by andesitic pyroclastic and metabasalts at the top of the SG (the Zhengjiaya Formation) (Fig. 1), where the metabasalts have a chemistry consistent with an island-arc source^33,34^ pointing to an active continental margin setting for the SG in the late Mesoproterozoic Era. Late Mesoproterozoic Era island-arc-related volcanic rocks are found elsewhere on the Yangtze block^34,37^, and a late Mesoproterozoic-early Neoproterozoic ophiolite is emplaced just south (ca. 40 km) of the SG^38^, consistent with continent–continent collision and continental assembly. The SG is unconformably overlain by Neoproterozoic-aged sediments Machaoyuan Group (ca. 750–541 Ma), which deposit broadly on the Yangtze Block^32^.

### Field observations

**Fig. 2:**
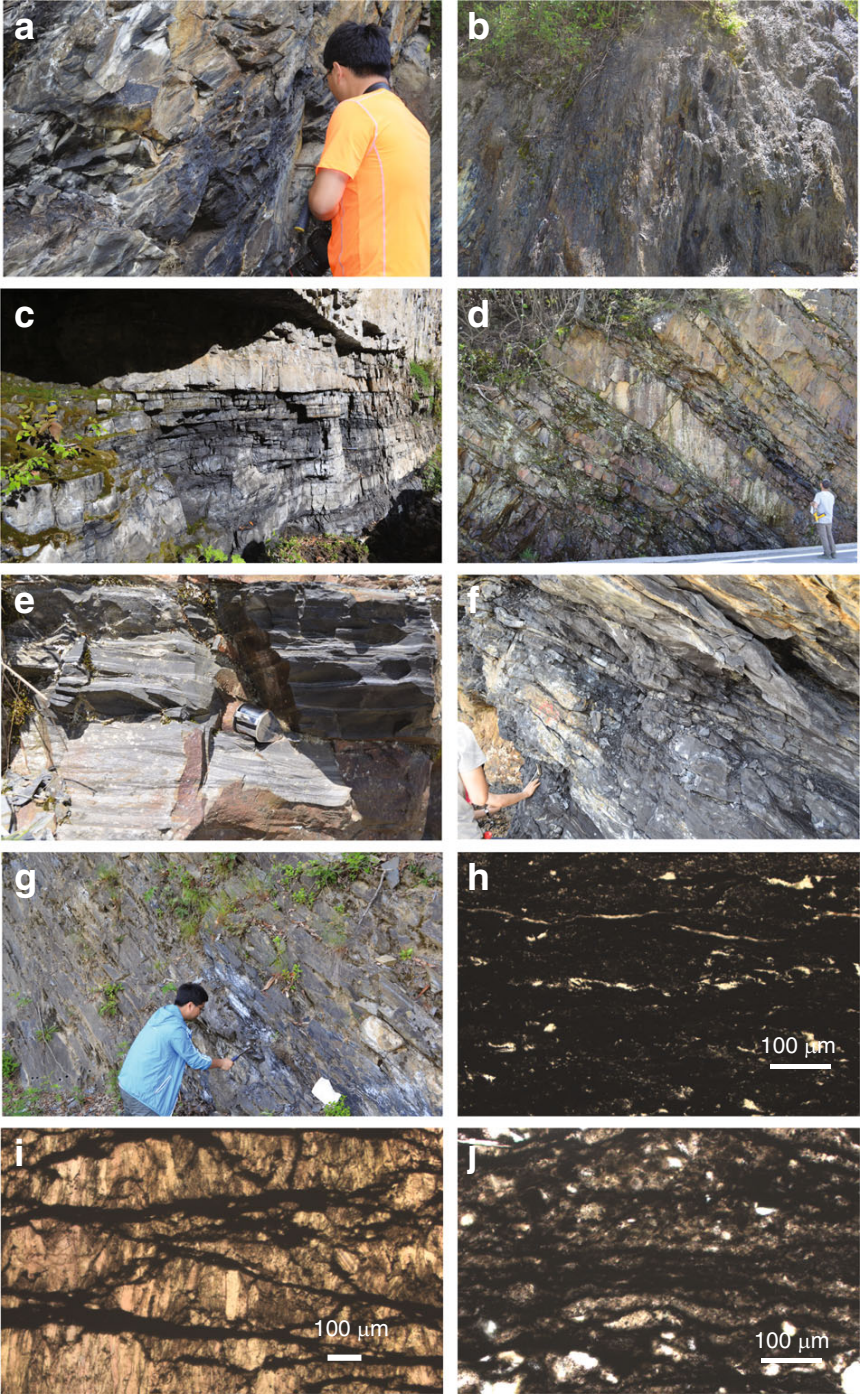
Outcrop photos and thin section photomicrographs of selected samples. **a** Outcrop photo of black shales from the Zhangjiaya Formation, **b** outcrop photo of shales from the Wenshuihe Formation, **c** alternating black shales and carbonates from the upper Taizi Formation, **d** alternating sandstones and black shales from lower in the upper Taizi Formantion than in photo, **d**, **e** close-up of sandstone unit from the upper Taizi Formation showing cross laminations that we interpret as evidence for a mass flow deposit, **f** a layer of massive black shale from the lower Taizi Formation, **g** shales from the Dayanping Formation, **h** thin section photomicrograph of black shale from the Zhengjiaya Formation (sample SZY-5), **i** thin section photomicrograph of organic matter layered carbonate-rich sediment from the upper Taizi Formation (sample TZ-1-3), **j** thin section photomicrograph of black shale from the lower Taizi Formation (sample TZ-26). Scale in photomicrographs, 100 μm

The Zhengjiaya Formation consists of a mix of black shales and cherts, with intervals of stromatolites and a conspicuous iron-ore interval. We sampled the black shales (Fig. 2a), and in thin section (Fig. 2h), they are fine grained, finely laminated with organic matter, and with quartz silicification. Where we sampled the Wenshuihe Formation, it consisted of finely laminated and silicified pyritic black shales (Fig. 2b). The Taizi Formation has a complex sedimentology. Stromatolites are found high up in section giving way to dolomite-black shales units (Fig. 2c), and then to alternating sandstone-black shale units (Fig. 2d). The sandstone units have both parallel and cross lamination (Fig. 2e), which we take as evidence for mass flow. Altogether, we interpret the sandstone-black shale intervals as black shale background sedimentation truncated by turbidite deposition. We sampled from both the dolomite-black shale interval (Fig. 2c) and the sandstone-black shale interval (Fig. 2d). In thin section, the sediments from the carbonate-rich interval are composed finely laminated organic matter in a mostly carbonate matrix cement (Fig. 2i). In the lower Taizi, the black shales are fine grained and finely laminated (Fig. 2f), where laminae are often disrupted by recrystallized quartz (Fig. 2j). The Dayanping Formation, the lowermost formation we sampled, is dominated by finely laminated dolostones in the lower part, ranging to finely laminated black shales in the upper regions. We sampled the black shales (Fig. 2g). All of our samples were taken after removing weathered materials to expose as fresh a surface as possible. Neither during our sampling nor during our petrographic observations of thin sections did we observe evidence for hydrothermal veins or other evidence for fluid flow or hydrothermal alteration.

### Data results

**Fig. 3:**
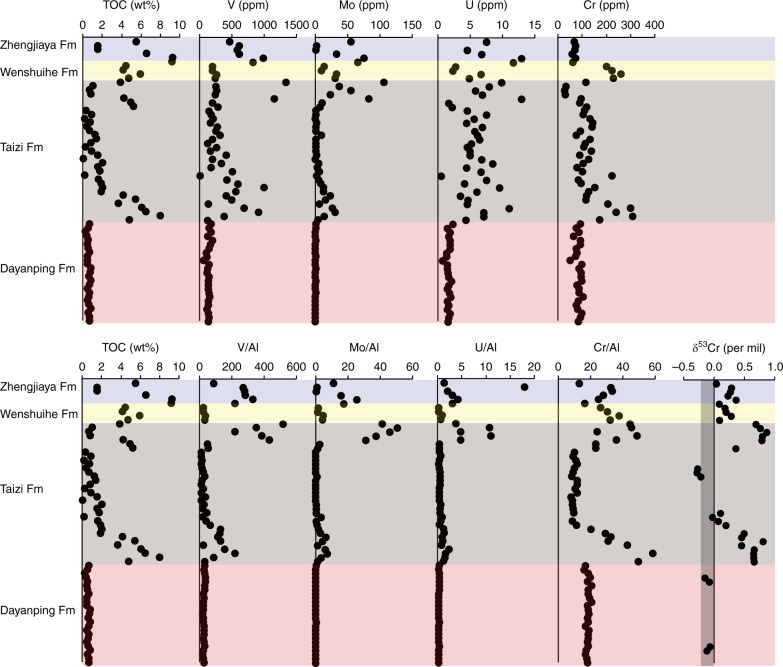
Geochemical data from the Shennongjia Group. Samples are plotted sequentially and relative to stratigraphic position (from top to bottom), but not as function of exact stratigraphic position, but rather by sample number (1, 2, 3, etc.). Therefore, the depth trends in the figure and do not reflect actual placement in the stratigraphy (see Fig. 1). The gray vertical bar in the δ^53^Cr plot represents the crustal average value^39^

**Fig. 4:**
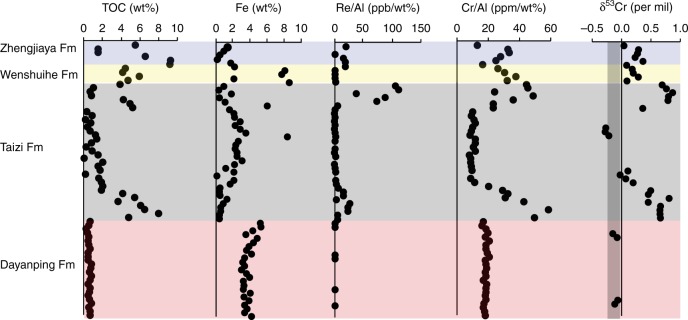
The isotopic composition of chromium (δ^53^Cr) compared with organic carbon (TOC) concentration, Fe concentration, Re/Al, and Cr/Al. As in Fig. 3, samples are plotted sequentially and relative to stratigraphic position (from top to bottom), but not as function of exact stratigraphic position. The gray vertical bar in the δ^53^Cr plot represents the crustal average value^39^

The black shales of the Zhengjiaya, Wenshuihe, and Taizi Formations have organic carbon concentrations (TOC) of up to 8–10 wt%, while the Dayanping Formation has much lower TOC values in the range of 0.33–0.87 wt% (Fig. 3, Supplementary Table 1). The TOC-enriched shales also house high concentrations of the redox-sensitive trace metals uranium (U), vanadium (V), molybdenum (Mo), and rhenium (Re), while the Dayanping Formation does not (Figs. 3, 4 and Supplementary Table 1). We also see enrichments in Cr concentration in the same intervals where TOC and the other redox-sensitive trace metals are enriched (Figs. 3, 4). Highly ^53^Cr-enriched chromium is found in those intervals with elevated trace metal concentrations, but especially those intervals with chromium concentration enrichments and elevated TOC. Values of δ^53^Cr range up to 0.9‰ in both the upper and lower intervals of the Taizi Formation (Fig. 3). In the Zhengjiaya and Wenshuihe Formations, the δ^53^Cr enrichments are not as great as in the Taizi Formation, but still, they reach values of 0.36‰ and 0.29‰, respectively (Fig. 3 and Supplementary Table 1), both well above the crustal average of −0.12 ± 0.1‰^39^.

**Fig. 5:**
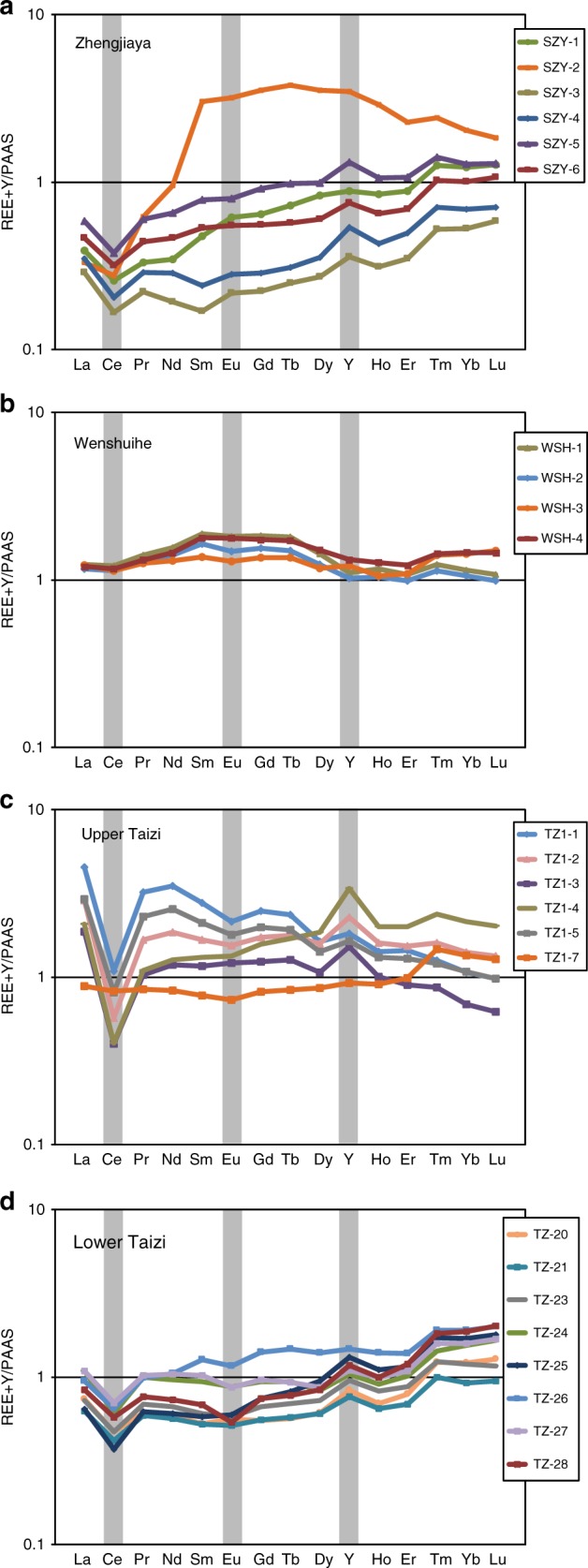
Rare earth element plus yttrium patterns for samples with elevated δ^53^Cr from the Shennegjia Group normalized against post Archean average shale (PAAS). The gray boxes highlight the elements Ce, Eu and Y . **a** Zhengjiaya formation, **b** Wenshuihe formation, **c** upper Taizi formation, **d** lower Taizi formation. Data are found in Supplementary Table 2

Low TOC portions of Taizi Formation, as well as the low TOC Dayanping Formation, show little or no enrichment in redox-sensitive trace metal concentration, and contain chromium with an isotopic composition within the crustal average range (Fig. 3 and Supplementary Table 1). Rare earth element (REE) plus yttrium concentrations normalized to post Archean average shale (PAAS)^40^ are plotted in Fig. 5 for sediments with elevated δ^53^Cr.

## Discussion

The combined enrichments of the redox-sensitive elements V, Mo, U, and Re in the TOC-rich sediments of the Zhengjiaya, Wenshuihe, and Taizi Formations (Figs. 3, 4) are typical for sediments deposited under anoxic water-column conditions^41^. Indeed, the enrichments observed in SG sediments either match or exceed those reported in other Mesoproterozoic black shales (Table 1). The trace metal enrichments are also comparable to (or even greater in some cases) than those observed in modern sediments from anoxic settings such as the Black Sea, the Saanich Inlet, and the Peruvian upwelling zone (Table 1). Therefore, we conclude that water-column anoxia was likely during the deposition of portions of the Zhengjiaya, Wenshuihe, and Taizi Formations, but without Fe speciation results, which are unreliable for outcrop samples, we cannot be certain as whether the dominant water-column chemistry was sulfidic, ferruginous, or nitrogenous. The Dayanping Formation sediments we sampled were laminated black shales, but with their relatively low TOC concentrations and lack of redox-sensitive trace metal enrichments (Figs. 3, 4, Table 1), the water chemistry is unclear and was possibly oxic.

**Table 1:** Maximum trace metal concentrations and enrichments Mesoproterozoic shales and modern sediments from anoxic environments

Formation	age	Mo	Mo/Al	Mo/Ti	V	V/Al	V/Ti	U	U/Al	U/Ti	Cr	Cr/Al	Cr/Ti	ref
Zhengjiaya	1100	76	25	490	990	330	6400	13	4.3	83	76	34	650	this study
Wenshuihe	1200	36	33	23	270	40	190	6.7	1	4.4	260	38	170	this study
Taizi	1330	107	51	530	1340	520	6700	13	11	103	310	72	880	this study
Dayanping	1400	1.8	0.34	3.6	200	41	370	2.4	0.47	4.5	107	21	270	this study
Velkerri	1400	120	37	420	560	160	2000	12	4.2	48	48	14	160	^65, 30^
Xiamaling	1390	48	8.2	150	820	130	2300	14	2.3	39	65	11	170	^30, 50^
Black Sea (unit 1)	0	121	32	680	180	56	1160	16	5.4	110	63	13	280	^66^
Peru (OMZ)	0	96	24	95	410	130	2500	34	9.7	202	150	50	1100	^67^
Cariaco Basin	0	84	14	420	210	35	1000				135	18	350	^49, 68^
Saanich Inlet	0	69	13	200	150	31	510				90	18	270	^69^
Crustal average	0	1.1	0.13	2.9	97	12	260	2.7	0.33	7.1	92	11	240	^70^

*Metal concentrations presented in ppm. †Metal ratios presented in ppm/wt%

Patterns of PAAS-normalized REE plus yttrium (Fig. 5) show a mix of lithogenic and authigenic contributions. For example, the Wenshuihe Formation shows a dominantly lithogenic signal (Fig. 5b). In contrast, negative Ce anomalies (Supplementary Figure 1), small negative or negligible Eu anomalies, positive Y anomalies, and a tendency toward light REE depletions relative to heavy REE are observed in the Zhengjiaya Formation (Fig. 5a) for all but one sample and in the lower Tazi Formation (Fig. 5d). These signatures are characteristic of oxygenated surface seawaters^42^. With a negative Ce anomaly, a positive Y anomaly, but no obvious heavy REE enrichment, the upper Taizi Formation (Fig. 5c) seems to display a mixture of a seawater and a lithogenic signal. Perhaps surprising is the negative Ce anomaly, as this might not be expected for waters in an anoxic basin, and is not found, for example, in the deep anoxic waters of the Black Sea^43^. However, negative Ce anomalies are found in carbonate-rich sediments depositing in anoxic waters of the Arabian Sea^44^, and in sediments from ancient Ordovician to early Silurian anoxic settings from Scotland^45^. Therefore, negative Ce anomalies can be preserved in sediments depositing in anoxic water columns. Overall, REE plus Y patterns reinforce the conclusion that sediments from the SG with ^53^Cr enrichments exhibit a strong authigenic component deposited from ancient seawater.

Chromium concentrations are enriched in the same intervals where other redox-sensitive trace metals are enriched (Figs. 3,4). Such enrichments might be expected as chromium is very redox sensitive, and the reduction of Cr(VI)O_4_^2−^ to particle-reactive Cr(III) species has been documented in the water column of modern nitrite-enriched oxygen-minimum zones (OMZs)^46^, as well as in the sulfidic waters of Saanich Inlet, British Columbia^47^. One might also expect similar behavior in the modern anoxic Black Sea water column. Indeed, concentration depth profiles reveal the apparent reduction of CrO_4_^2−^ to Cr(III) and Cr removal from solution in the oxygen-free, sulfide-free chemocline region^47^. However, counter to expectations, dissolved chromium accumulates again in the underlying sulfidic waters^47^. This geochemical behavior is not well understood, but Cr(III) phases may be scavenged in the chemocline by adsorption onto Mn oxides, with subsequent liberation to solution in the underlying sulfidic waters as Mn oxides are reduced^47^. The liberated Cr(III) may then be stabilized in solution by dissolved organic complexes^47^. Overall, and despite the sulfidic waters, chromium does not apparently accumulate above crustal average concentrations in Black Sea sediments (Table 1). In contrast, chromium does accumulate in the OMZ sediments of the Peru margin, in sediments of the sulfidic Cariaco Basin, and possibly also in Saanich Inlet sediments, but to an extent much less than in Cariaco Basin or Peru Margin OMZ sediments (Table 1). Therefore, while Cr is highly redox active and accumulates in the sediments of many modern anoxic water-column settings (Table 1), it does not accumulate in all of them (see also ref.^48^). Clearly, further study is required to understand what ultimately controls the accumulation of chromium in anoxic marine settings.

**Fig. 6:**
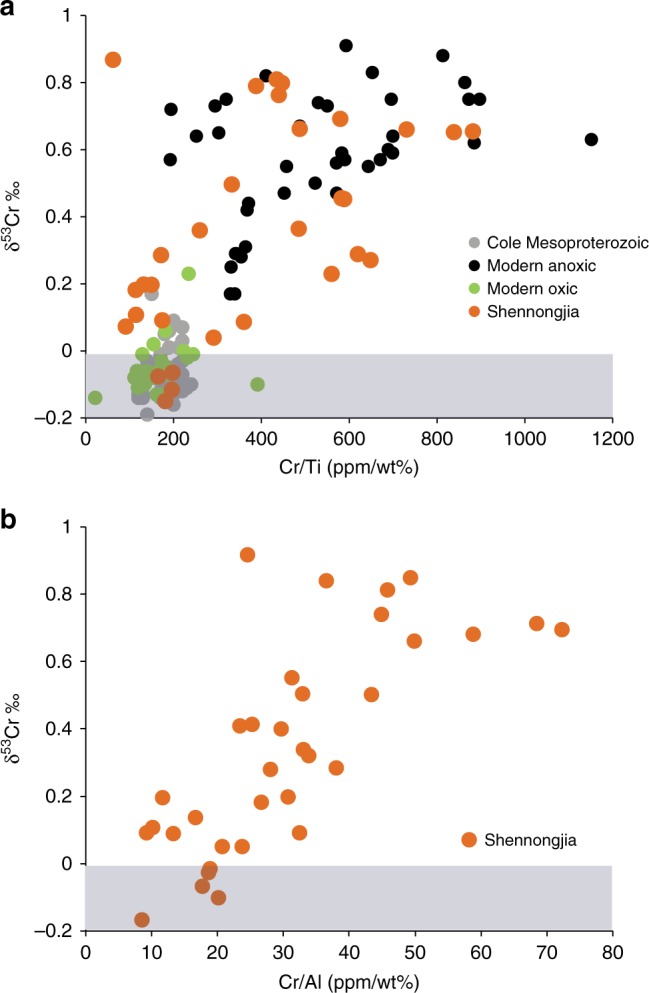
The isotopic composition of chromium (δ^53^Cr) compared to chromium enrichment. **a** Enrichment relative to Ti (Cr/Ti). Samples from the Shennongjia Group are given in orange circles, while modern sediments deposited in anoxic settings are given in black circles and modern oxic settings are given in green circles. Mesoproterozoic-aged shales from ref.^30^ are given in gray circles. **b** Enrichment relative to Al (Cr/Al) for samples of the Shennongjia Group. Cr/Al data are not available for other modern and ancient sediments. The shaded horizontal bar represents the δ^53^Cr range for crustal values^39^. All data and references are summarized in Supplementary Table 3

Unlike previous analyses of Mesoproterozoic black shales^30^, we observe large enrichments in ^53^Cr in SG sediments. The enrichments follow those in modern sediments depositing in anoxic waters, both in overall magnitude and in relationship to Cr concentration enrichment (Fig. 6a). Thus, as for sediments in modern anoxic environments^49^, chromium isotopes in SG sediments reflect the addition of a ^53^Cr-enriched authigenic component to a lithogenic component with low Cr/Al, low Cr/Ti, and low values of δ^53^Cr (Fig. 6a, b). Furthermore, as trends in δ^53^Cr versus Cr concentration for SG sediments are similar to those from modern sediments, Mesoproterozoic Era seawater chromate was also likely enriched in ^53^Cr by similar amounts to today. If chromate in seawater during SG deposition was less ^53^Cr-enriched than modern seawater, we would expect a shallower trend in Cr concentration versus δ^53^Cr, and if ancient seawater was more ^53^Cr-enriched, we would expect a steeper trend.

Present seawater displays δ^53^Cr values ranging from about 0.3 to 1.6‰^25,26^. Modern seawater chromate also has a typical surface seawater ^53^Cr enrichment attributed to a fractionation of about −0.8‰ associated with chromium removal from these surface waters^25,26^. The mechanisms of chromium removal are not clear, but may be associated with a limited biologically induced reduction of Cr(VI) to Cr(III) in the productive oxygenated surface waters^26^. If a similar fractionation applied to Cr removal into anoxic SG shales, then the most enriched δ^53^Cr shale values of 0.3–0.85‰ (Fig. 3) imply removal from seawater with chromate δ^53^Cr values of 1.1–1.65‰. This range in δ^53^Cr values is similar those in the upper 500 m of modern seawater^26^.

Previously analyzed Mesoproterozoic-aged black shales from the Arctic Bay Formation (1100 Ma), the Xiamaling Formation (1390), and the Velkerii Formation (1400) generally lack ^53^Cr enrichments^30^ (Fig. 6a). In contrast to many of the SG shales, previously analyzed Mesoproterozoic-aged shales also lack obvious chromium concentration enrichments (Fig. 6a), implying a minimal authigenic chromium component, possibly explaining their lack of ^53^Cr enrichment^50^. Variability in the extent of authigenic chromium enrichment from anoxic Mesoproterozoic-aged depositional environments would be consistent with observations from modern anoxic environments, where, as discussed above, chromium concentration enrichments are quite variable (Table 1). As also noted above, the reasons for this variability are not well understood, but could relate to the extent to which chromium (likely as Cr(III)) is re-mobilized to the anoxic water column as carrier phases are reduced and as organic ligands accumulate to complex the liberated dissolved chromium^47^.

Our findings of highly ^53^Cr-enriched Mesoproterozoic-aged shales also contrast with observations from the oolitic ironstones of the 1450 Ma Sherwin Formation, where isotopically fractured chromium was not found^8^. It is unclear why the Sherwin Formation does not contain fractionated chromium. One possibility is that the Sherwin Formation oolites contain only a small compliment of authigenic chromium. This is suggested by the linear relationship between chromium and titanium concentrations in these ironstones^51^, where titanium provides a measure of the lithogenic component. As another possibility, the reduction of Cr(VI) to Cr(III), with subsequent immobilization into iron oxides, has an associated fractionation of about 1.8‰, where the iron-oxide-hosted Cr(III) is ^53^Cr depleted relative to the Cr(VI) precursor^52^. Since oolites typically form in high-energy environments, they should experience minimal reservoir isotope effects as authigenic chromium is precipitated. This means that high fractionations should be expressed, and that the isotopic composition of authigenic chromium in the oolitic ironstones should be less, and possibly much less, than the seawater supplying the chromium^52^. Therefore, if the chromium in the Sherwin Formation is authigenic, it likely formed from a chromate reservoir with a more ^53^Cr-enriched isotopic composition.

Our results indicate that from 1080 to 1330 Ma, Mesoproterozoic seawater contained chromate with a δ^53^Cr similar to today, but as mentioned above, oxidative weathering may not be the only process producing chromium isotope effects. Isotopic fractionation can also occur as Cr(III) is solubilized by a variety of organic ligands including products of organic matter fermentation (succinate, acetate, and citrate) as well as oxalate^53^. Ligand dissolution liberates Cr(III) both enriched and depleted in ^53^Cr, and the extent of fractionation decreases with greater extents of dissolution. How these processes may have impacted the ancient chromium cycle is unclear. Indeed, the potential importance of ligand-driven Cr(III) dissolution on land before the development of a substantial terrestrial biosphere is uncertain, but possibly limited. Organic complexation, however, could have promoted some remobilization of Cr(III) in organic-rich marine sediments, where fermentation products accumulate^53^. Therefore, the TOC-rich sediments of the SG could have been a source of ligand-complexed Cr(III) to seawater, but with an uncertain magnitude and uncertain isotope effects. Even so, ligand-driven Cr(III) dissolution does not explain the enrichments of chromium concentration in SG sediments and how these enrichments would be associated with ^53^Cr enrichments. Also, as highlighted in ref.^53^, the process of ligand complexation is unlikely to alter our view that substantial positive δ^53^Cr values in marine sediments reflects the oxidative weathering of chromium on land^53^.

The serpentinization of ultramafic rocks produces H_2_O_2_ that can oxidize Cr(III) phases^54^. The process of H_2_O_2_ oxidation leaves a ^53^Cr-enriched signal in the serpentinized materials^55,56^, where greater ^53^Cr enrichment correlates with greater chromium loss^56^ and with greater indications of rock alteration^55^. The processes generating this isotope effect are unclear, but one possibility is that serpentinization liberates isotopically depleted chromium, leaving ^53^Cr-enriched chromium behind in the altered rocks^55,56^. Another possibility is that early stage serpentinization produces oxidative loss of Cr(III) with no isotope effect, followed by the addition of ^53^Cr-enriched seawater chromium during late-stage seawater alteration^56^. Such a scenario, however, does not explain why the greatest ^53^Cr enrichments in ultramafic rocks are found in samples most depleted in chromium concentration^56^. In any event, these processes are unlikely to have influenced the δ^53^Cr in our SG samples as these samples are all typical marine sediments and could not have supported serpentinization reactions. Furthermore, as noted above, serpentinization seems to liberate chromate to solution with δ^53^Cr values less than or equal to the crustal average. Thus, even if serpentinization occurred locally, as was possible during the deposition of the Zhengjiaya Formation (with possibly contemporaneous ophioliote emplacement, see above), the isotopic composition of any liberated chromate would have been ^53^Cr depleted (or the same as average rock), and could not explain the ^53^Cr-enriched values of the contemporaneous SG sediments.

We collected and analyzed outcrop samples and must be mindful of any possible weathering effects on our chromium isotope results^56,57^. Indeed, ^53^Cr isotope enrichments have been observed in the most weathered portions of the organic-rich 365 Ma New Albany Shale (NAS)^56^ and in weathered portions of the Mesoarchean (2950 Ma) Ijzermijn iron formation (IF)^57^. The ^53^Cr enrichments are attributed to the immobilization of ^53^Cr-enriched chromate from the weathering fluids by either adsorption onto Fe oxides (NAS) or by reduction with Fe (II) phases, followed by incorporation into iron oxides (IF). The TOC-enriched NAS is closest in sediment type to TOC-rich shales we analyzed, and in the NAS, δ^53^Cr correlates positively with enrichments in chromium concentration. In the NAS, ^53^Cr enrichments are also found together with the complete loss of the redox-sensitive element Re during the extreme weathering of the shale.

In SG sediments, by contrast, elevated concentrations of Re are typically found in the same horizons supporting elevated δ^53^Cr, elevated chromium concentrations and elevated TOC (Fig. 4). Therefore, the sediments of the SG are not as weathered as those of the NAS. We also see the strongest ^53^Cr enrichments in the Fe-poor sediments of the lower Taizi Formation (Fig. 4), and overall we see a negative trend between δ^53^Cr and total Fe concentration (Supplementary Fig. 2). This observation is inconsistent with Fe acting to immobilize ^53^Cr-enriched chromium during weathering. The SG sediments are also of high thermal maturity, so organic matter is an unlikely reductant in the contemporary conversion of chromate to immobile Cr(III)^56^. Overall, intervals of elevated δ^53^Cr in SG sediments are not compatible with an origin from modern weathering. These intervals do, however, correlate with high concentrations of TOC and enrichments in redox-sensitive trace metals. Therefore, elevated δ^53^Cr values in our SG sediments are most parsimonious with an early diagenetic origin from a ^53^Cr-enriched seawater source derived from oxidative weathering of Cr(III) on land.

**Fig. 7:**
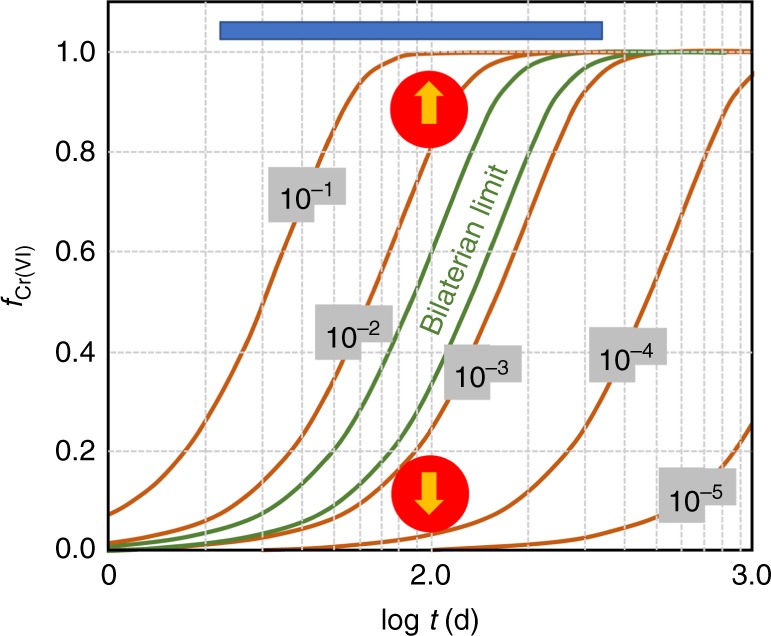
Oxygen model results. Results are redrawn from ref.^8^ including interpolated results for oxygen levels of 10^−2^ PAL and 10^−4^ PAL, not presented in the original figure. The *x* axis presents soil water residence time, and the blue bar on top represents the range in modern soils. The *y* axis represents the fraction of the original Cr(III) in the soil oxidized to Cr(VI) and the contours represent different levels of atmospheric oxygen in fraction of PAL. The lower-limit range of oxygen requirements for bilaterian animals with a simple circulatory system^64^ is constrained between the green lines. The lower red circle represents the maximum atmospheric oxygen concentration as constrained by Planavsky et al.^8^, whereas the upper red circle represents the minimum atmospheric oxygen estimate of the present study

**Fig. 8:**
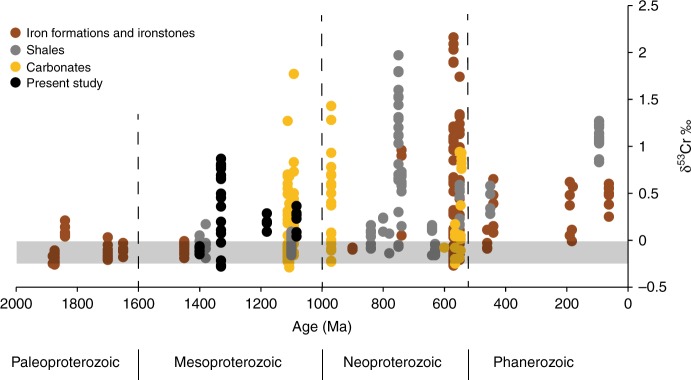
Compilation of sediment δ^53^Cr values over the last 2000 Ma. The gray horizontal bar in the δ^53^Cr represents the crustal value^39^. All data and references are found in Supplementary Table 3

Previous model results suggest that at lack of δ^53^Cr enrichment in Mesoproterozoic sediments, and the absence of oxidative weathering of Cr(III) from soils that these results imply, is consistent with <0.1% PAL of atmospheric oxygen^8^. This model result arises from a typical soil weathering environment, a soil water residence time of about 100 days, and with <20% conversion of Cr(III) to Cr(VI) during weathering^8^ (Fig. 7; see details in Supplementary Discussion, Oxygen concentration model). In contrast, a significant conversion of Cr(III) to Cr(VI) during weathering (>80%, Fig. 7), as is more compatible with our results, occurs at atmospheric oxygen levels of >1% PAL. Therefore, with available model constraints, our finding of highly fractionated Cr in Mesoproterozoic SG shales is consistent with at least 1% PAL atmospheric oxygen. This oxygen estimate is in line with independent minimum estimates of >4% PAL as required to explain the geochemical record of units 1 and 3 of the ca. 1390 Ma Xiamaling Formation of the North China Block^58,59^. Our current results are further reinforced by highly fractionated Cr extracted from several carbonate deposits in the time window from 970 to 1112 Ma^31^ (Fig. 8).

In contrast to previous reports^8,30^, our findings show that enriched δ^53^Cr is a persistent feature of the Mesoproterozoic Era marine geochemical record (Fig. 8), providing a minimum, rather than maximum, estimate of atmospheric oxygen levels. Indeed, our results, combined with previous observations of δ^53^Cr in carbonates^31^, and other minimum estimates of atmospheric oxygen levels^58,59^, lead us to conclude that oxygen concentrations of >1% PAL (with >4% PAL at 1390 Ma) were a persistent feature of the Mesoproterozoic Era atmosphere, at least from 1390 Ma and onward. As noted above, oxygen concentrations in this range were likely sufficient to fuel the metabolism of early crown-group animals. Therefore, the geochemical record of chromium isotopes reveals that for prolonged periods of time the atmosphere contained sufficient oxygen to fuel animal metabolism long before animals evolved.

We do not imply that the history of atmospheric oxygen is irrelevant to the history of animal evolution. It seems clear that the Mesoproterozoic ocean experienced greatly expanded anoxia compared to today^60–62^, requiring that atmospheric oxygen concentrations were much less than current levels. Furthermore, most modern animals, including both vertebrates and invertebrates, could not survive at oxygen levels as low as 1–4% PAL^63^. Therefore, it seems likely that increases in atmospheric oxygen beyond Mesoproterozoic levels were required to support many animals with the physiological requirements of those living today. Understanding the history of these increases will be critical to fully understand relationships between the history of atmospheric oxygen and animal evolution.

## Methods

### Trace metals, major elements, and TOC

Samples were crushed to powders with a diameter <74 µm. Each rock powder was oven-dried overnight at 105 °C. An aliquot of 2.0 g of sample was precisely weighed and then ignited for 20 min at 1100 °C in a Pt(5%)Au crucible and then re-weighed to determine the loss on ignition (LOI). Following this, 0.5 g of each ignited sample was precisely weighed and mixed with Li_2_B_4_O_7_–LiBO_2_–LiF (4.5:1:0.4, wt %) in an unignited sample-to-flux ratio of 1:9. The ignited powder and the flux were fused in a porcelain crucible for 20 min at 1100 °C in a muffle furnace, where the molten mixture was constantly swirled to completely disperse the flux. Cooled samples were re-weighed, and any weight lost was made up by adding extra flux. Samples were fused for a second time over a Meker burner, swirling the molten mixture during heating to ensure homogenization, and then cast on a graphite mold on a 250 °C hotplate. The sample was then pressed with an aluminum plunger to create a flattened disk. Major element concentrations were measured by X-ray fluorescence (Philips Electronics, PW2404) to determine the concentrations of major element oxides. Accuracies were tested with shale standard material (GBW 03014) and whole-rock standard materials (GBW 07109–07112). The relative standard deviation of major element concentrations was <1.0%.

Homogeneous dried powders of whole-rock samples were also prepared for trace metal element analysis with high-resolution ICP-MS (Finnigan MAT, Element I). Powders were dissolved using a tri-acid digestion involving HNO_3_, HClO_4_, and HF. First, 0.5 g of each ignited powder was precisely weighed and transferred to a teflon crucible. Then, 7–8 ml concentrated HF and 5 ml of 50% HNO_3_ were added, and the sample was left on a hotplate to dissolve overnight. The samples were boiled to near dryness on a 250 °C hotplate. After a few minutes of cooling, 7–8 ml concentrated HF were added again and boiled to near dryness. Then, 5 ml of 50% HNO_3_ were added to each dry sample and left on the hotplate to dissolve overnight. Cooled samples were re-boiled the next day with 1 ml HClO_4_ until the white smoke completely disappeared. Then, cooled samples were heated with 5 ml of 50% HNO_3_ until the solution became transparent. Following this, each sample was diluted with 5% HNO_3_ to 50 ml. Trace element concentrations were measured for all samples from the diluted solutions and the accuracies were tested with the shale standard (GBW 03014) and the whole-rock standard (GBW 07109–07112). The relative standard deviation of the trace element analyses was <1.5%.

The concentrations of organic carbon (TOC) were determined on crushed rock powders, after acid treatment to remove carbonates, by combustion on a LECO CS-230HC with an uncertainty of <1%.

### Chromium isotopes, sample preparation

Bulk samples were powdered in an agate disk mill, and analyses were performed on 5–30 mg of powdered black shale and carbonate-rich samples, which were attacked by a concentrated HF-aqua regia mix on a hotplate at 120 °C overnight.

### Chromium separation

Samples were spiked with a ^50^Cr–^54^Cr double spike^39^ aiming for ^50^Cr/^52^Cr in the sample-spike mixture of between 0.15 and 0.75. The bulk samples were spiked during the HF-aqua regia dissolution step, while the leachates where spiked after the leaching exposure. Spike-sample homogenization was assured by renewed treatment of respectively dried down fractions by aqua regia. Chromium was separated in two steps using ion chromatographic separation schemes on respective extraction columns.

The first step used a pass over anion exchange resin-loaded column. The spiked samples were re-dissolved in ca. 18 ml of 0.1N HCl together with 0.5 ml of a freshly prepared 1N ammonium persulfate ((NH_4_)_2_S_2_O_8_) solution, which acted as an oxidizing agent. The sample solutions, contained in 23 ml Savillex Teflon beakers, were placed on a hotplate at 130 °C for 1 h to ensure full oxidation of Cr(III) to Cr(IV). After the samples cooled to room temperature, they were passed through anion exchange columns (BioRad) loaded with 2 ml of pre-cleaned Dowex AG 1 × 8 anion resin (100–200 mesh). The matrix was washed out with 10 ml of 0.2N HCl, then with 2 ml of 2N HCl and finally with 5 ml of pure H_2_O (18 MΩ MilliQ), before Cr was collected through reduction with 6 ml 2N HNO_3_ doped with a few drops of 5% H_2_O_2_. The so-stripped Cr-bearing solution was then dried down at 130 °C.

The second step used pass over cation exchange resin-loaded columns. For this, the Cr-bearing samples from the anion columns were re-dissolved in 100 μl of concentrated HCl and diluted with 2.3 ml ultrapure MilliQ water. This solution was added to the extraction columns loaded with 2 ml of pre-cleaned Dowex AG50W-X8 cation resin (200–400 mesh). The final Cr-bearing liquid cut was dried down at 130 °C, ready to be loaded for Cr isotopic analysis on the thermal ionization mass spectrometer.

### Thermal ionization mass spectrometry (TIMS)

The Cr isotope measurements were performed on an IsotopX, Ltd. IsoProbe T thermal ionization mass spectrometer (TIMS) equipped with eight Faraday collectors that allow simultaneous collection of the four chromium beams (^50^Cr^+^, ^52^Cr^+^, ^53^Cr^+^, and ^54^Cr^+^) together with interfering ^49^Ti^+^, ^51^V^+^, and ^56^Fe^+^ masses.

The separated Cr residues were loaded onto outgassed Re filaments using a loading solution consisting of 1 μl of 0.5N H_3_PO_4_, 2.5 μl silicic acid, and 0.5 μl of 0.5N H_3_BO_3_. The samples were analyzed at temperatures between 1050 and 1250 °C maintaining ^52^Cr beam intensities of between 0.5 and 1 V. One run consisted of 120 cycles, and every sample was run at least twice. The final δ^53^Cr values of the samples were determined as the average of the repeated analysis and are reported in ‰ with ±standard deviation (2*σ*) relative to the international standard reference material NIST SRM 979 as

1 $$\delta ^{53}{\mathrm{Cr}}\left( \textperthousand \right) = \left( {\,{}^{53}{\mathrm{Cr/}}\,{}^{52}{\mathrm{Cr}}_{sample}} \right){\mathrm{/}}\left( {\left( {\,{}^{53}{\mathrm{Cr/}}\,{}^{52}{\mathrm{Cr}}_{NIST\,SRM\,979}} \right) - 1} \right) \times 1000$$

The within-run two standard errors of the measurements reported in this study were consistently ≤0.06‰. The external reproducibility was determined using average δ^53^Cr values of double-spiked NIST SRM 979 measured under the same conditions as the samples on the IsoProbe T. The composition of the NIST SRM 979 showed an offset of −0.04 ± 0.11‰ (2*σ*; *n* = 32) compared to the 0‰ certified value of this standard. This offset stems from the original calibration of our double spike relative to the NIST 3112a Cr standard, and the observed offset of 0.04‰ was added to the raw δ^53^Cr results to account for this discrepancy. Procedural Cr blanks remained at <15 ng total Cr. These levels are negligible compared to 0.5–1 μg of Cr processed in the samples. Therefore, no blank corrections were undertaken.

### Data availability

All data reported in this paper are available in the Supplementary Information.

## Electronic supplementary material

Supplementary Information
