# Supplementary material for: Highly fractionated chromium isotopes in Mesoproterozoic-aged shales and atmospheric oxygen
Source: Nat Commun. 2018 Jul 20;9:2871. doi: 10.1038/s41467-018-05263-9 (PMC6054612; doi:10.1038/s41467-018-05263-9)
Supplement: Supplementary file 1 — Supplementary Information [file 41467_2018_5263_MOESM1_ESM.pdf]

Highly fractionated chromium isotopes in Mesoproterozoic-aged shales and atmospheric oxygen

Canfield et al.

Supplementary Discussion

Oxygen concentration model

Estimates of atmospheric oxygen concentrations from chromium isotope systematics are based on the results of a kinetic oxidation model as presented in reference ([1]). The model recognizes that the proximal oxidant for Cr(III) to Cr(VI) is Mn oxides and not oxygen. Thus, the model combines the kinetics of the inorganic reaction of Cr(III) phases with MnO_2 phases with the kinetics of Mn^{2+} reoxidation to MnO_2 with oxygen, both biological and inorganic, with biological kinetics preferred. The model considers a range of time scales relevant for water retention in soils, and thus the time scale for the reactions to occur. The model results presented in the main text of reference ([1]) are reproduced in Figure 7. Based on these results reference ([1]) argued that low chromium isotope fractionations, representing minimal Cr(III) oxidation on land, would be preserved at < 0.1% PAL of atmospheric oxygen. Although not explicitly stated in reference ([1]), such maximum pO_2 estimates oxidize <20% of the available Cr(III) to Cr (VI) at a nominal soil water residence time of 100 days as indicated in Figure 7.

As mentioned in the main text of the present study, our results demonstrate that high fractionations are preserved in some Mesoproterozoic-aged sediments implying highly fractionated Cr(VI) in Mesoproterozoic seawater, with values likely similar to seawater today. Thus, the weathering efficiency of Cr(III) to Cr(VI) was far more extensive than previously indicated[1], and may have been even similar to today. We assume that >80% oxidation of Cr(III) to Cr(VI) would generate these results, and this extent of Cr(III) oxidation is consistent with > 1% PAL as indicated on Figure 7. This estimate cannot be viewed as precise, but it is clear, as opposed to previous contributions, that our results provide a minimum estimate for atmospheric oxygen. In addition, this estimate must be considerably higher than the maximum estimates of previous contributions highlighting no fractionation of chromium in Mesoproterozoic-aged sediments. Thus, based on the

current understanding of the relationship between sediment chromium isotope fractionation and atmospheric oxygen, a minimum Mesoproterozoic oxygen level of > 1% PAL seems well justified.

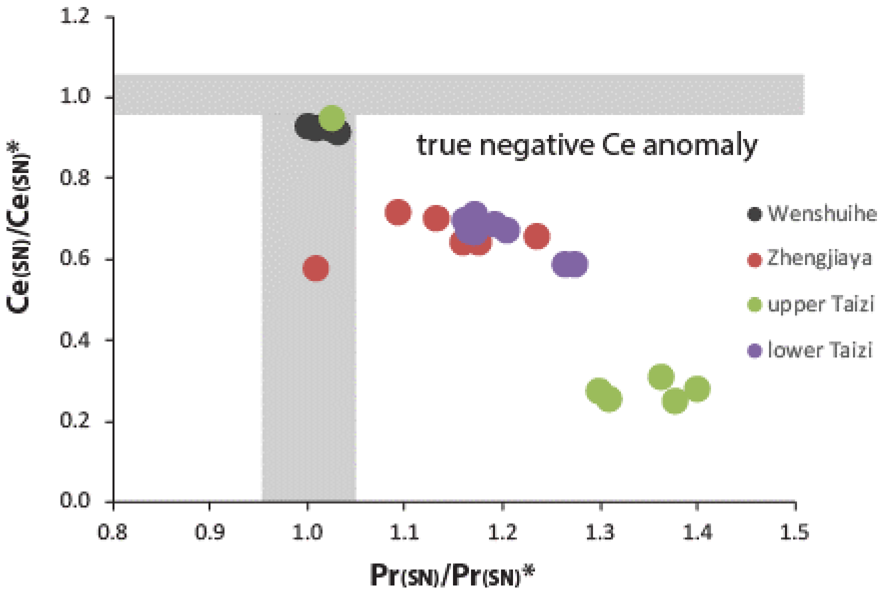

Supplementary Figure 1. Ce anomalies (Ce(SN)/(0.5 La(SN)+0.5Pr(SN)) plotted against Pr anomalies (Pr(SN)/(0.5Ce(SN)+0.5Nd(SN)) for sediments with enriched δ^{53}Cr values from the Shennengjia Group. The field indicating a true Ce anomaly is indicated[2].

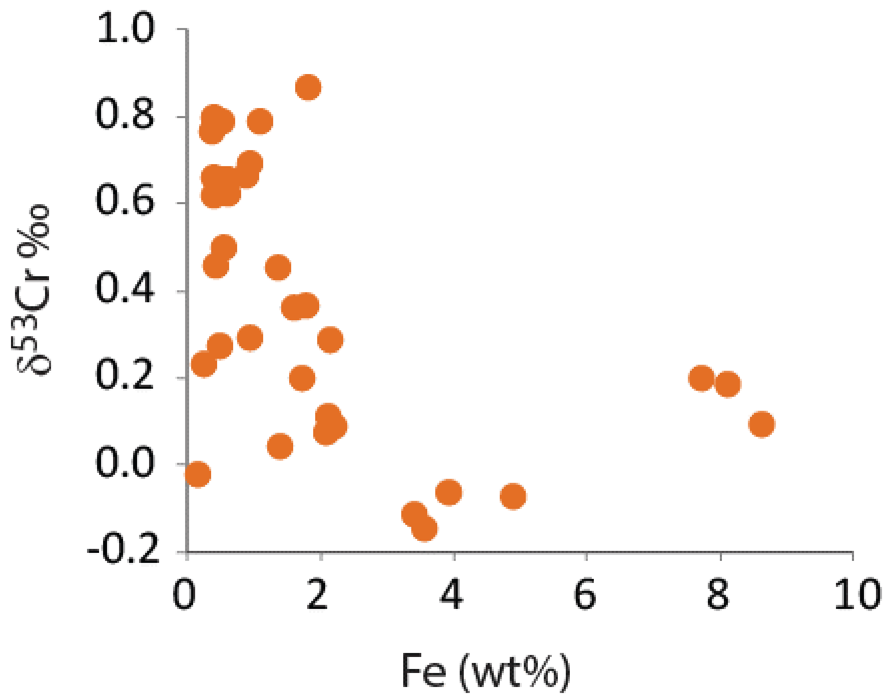

Supplementary Figure 2. Relationship between total iron and $\delta^{53}Cr$ for sediments of the SG.

Supplementary Table1. Geochemistry of Shennongjia Group sediments

Sample	Age Ga	Formation	Lithology	TOC wt%	$\delta^{53}Cr$	2s	n	Cr μg/g	Re ng/g	CaO wt%	Al_2O_3 wt%	TiO_2 wt%	Fe_2O_3 wt%	V μg/g	Cr μg/g	Mo μg/g	U μg/g
SZY-1	1.10	Zhengjiaya	Black mudstone	5.52	0.039	0.047	2	67.8	1	0.09	9.6	0.39	2.0	475	68	55.9	7.55
SZY-2	1.10	Zhengjiaya	Black mudstone	1.57	0.288	0.044	2	75.1	<2	4.91	4.3	0.20	1.37	623	75	2.4	41
SZY-3	1.10	Zhengjiaya	Black mudstone	1.55	0.270	0.048	2	70.5	2	0.05	3.9	0.18	0.69	586	71	0.8	4.63
SZY-4	1.10	Zhengjiaya	Black mudstone	6.60	0.229	0.045	2	60.2	33	0.29	4.1	0.18	0.36	618	60	33.8	6.83
SZY-5	1.10	Zhengjiaya	Black mudstone	9.31	0.364	0.047	2	75.8	56	0.85	5.7	0.26	2.54	996	76	76.2	13
SZY-6	1.10	Zhengjiaya	Black mudstone	9.26	0.086	0.043	2	62.5	70	0.46	7.1	0.29	3.17	835	63	65.8	11.7
WSH-1	1.20	Wenshuihe	Black shale-rich in iron	4.50	0.181	0.046	7	201	9	0.29	14.3	2.97	11.6	201	201	14.6	2.76
WSH-2	1.20	Wenshuihe	Black shale-rich in iron	4.19	0.197	0.042	1	224	8	0.25	13.8	2.85	11.0	204	224	10.4	2.39
WSH-3	1.20	Wenshuihe	Black shale	5.98	0.285	0.116	3	261	10	0.06	13.0	2.56	3.06	271	261	33.5	6.7
WSH-4	1.20	Wenshuihe	Black shale-rich in iron	4.77	0.091	0.054	5	230	13	0.35	13.4	2.22	12.3	245	230	30.8	4.95
TZ1-1	1.33	Taizi-upper	Black shale	3.93	0.691	0.052	2	116	273	13.75	4.9	0.34	1.34	1340	116	107.0	9.9
TZ1-2	1.33	Taizi-upper	Black argillaceous limestone	1.08	0.763	0.047	2	33.7	82	41.94	1.4	0.13	0.55	260	34	37.4	7.96
TZ1-3	1.33	Taizi-upper	Black argillaceous limestone	0.70	0.868	0.044	2	29.6	46	46.27	2.3	0.80	2.58	270	30	55.8	5.87
TZ1-4	1.33	Taizi-upper	Black argillaceous limestone	0.87	0.798	0.049	2	30.8	55	44.32	1.2	0.12	0.59	244	31	23.4	6.89
TZ1-5	1.33	Taizi-upper	Black lime mudstone	4.30	0.789	0.047	2	97.3	197	20.56	5.0	0.42	1.56	1170	97	83.0	13
TZ1-6	1.33	Taizi-upper	Black mudstone	5.01				92.7	23	0.23	7.4	0.64	8.68	203	93	10.6	1.78
TZ1-7	1.33	Taizi-upper	Black mudstone	5.24	0.359	0.061	2	121	8	0.09	9.8	0.78	2.29	293	121	7.6	2.3
TZ-1	1.33	Taizi-lower	Black shale-gray shale	0.34				110	5	0.06	20.0	1.07	3.00	152	110	1.1	4.65

TZ-2	1.33	Taizi-lower	Black shale	0.91				105	8	0.07	21.0	1.49	3.15	180	105	1.2	7.62
TZ-3	1.33	Taizi-lower	Gray shale	0.24				135	4	0.07	22.4	1.55	4.10	217	135	0.7	5.62
TZ-4	1.33	Taizi-lower	Black shale	0.80				145	7	0.07	22.5	1.34	3.29	178	145	2.5	4.49
TZ-5	1.33	Taizi-lower	Black shale-gray shale	0.43	0.269	0.059	2	142	4	0.10	25.6	1.44	4.10	280	142	1.0	6.9
TZ-6	1.33	Taizi-lower	Black shale	0.75	0.280	0.050	2	93.8	6	0.06	18.4	1.12	5.02	255	94	1.4	5.8
TZ-7	1.33	Taizi-lower	Black mudstone-rich in iron	1.30	0.216	0.051	2	78.7	16	0.13	17.4	1.48	12.0	317	79	10.2	6.18
TZ-8	1.33	Taizi-lower	Black shale	1.45				134	8	0.47	21.0	1.50	3.79	201	134	0.9	6.57
TZ-9	1.33	Taizi-lower	Black shale	0.88				115	<2	0.05	17.7	1.08	3.40	136	115	1.6	5.2
TZ-10	1.33	Taizi-lower	Gray shale	0.30				108	6	0.05	18.9	1.31	3.21	264	108	1.0	4.6
TZ-11	1.33	Taizi-lower	Black shale	0.92				139	5	0.07	21.5	1.36	3.62	172	139	1.7	5.04
TZ-13	1.33	Taizi-lower	Black shale	1.59				91.2	24	0.05	20.5	1.37	3.57	421	91	2.9	5.05
TZ-14	1.33	Taizi-lower	Green shale	0.10				128	<2	0.06	26.0	1.38	4.39	206	128	0.6	6.83
TZ14-1	1.33	Taizi-lower	Black shale	2.05				105	5	0.05	21.4	1.66	3.15	348	105	5.2	8.5
TZ-15	1.33	Taizi-lower	Black shale	1.56				80.1	8	0.06	15.6	1.17	1.70	188	80	3.5	4.51
TZ-16	1.33	Taizi-lower	Black shale	1.80	0.107	0.044	5	103	20	0.04	19.2	1.51	3.03	515	103	7.1	6.73
TZ16-1	1.33	Taizi-lower	Black sandstone	0.22	0.025	0.061	5	225	<2	0.05	1.1	0.08	0.22	13	225	2.2	0.614
TZ-17	1.33	Taizi-lower	Black sandstone	1.66	0.072	0.025	5	86.9	22	0.07	17.7	1.60	3.00	428	87	5.2	7.55
TZ-18	1.33	Taizi-lower	Black shale	1.97	0.197	0.038	5	98	30	0.05	15.8	1.10	2.45	597	98	9.0	4.13
TZ-19	1.33	Taizi-lower	Black shale	2.06				154	51	0.04	14.0	0.92	0.62	1005	154	13.6	9.62
TZ-20	1.33	Taizi-lower	Black shale	1.93	0.496	0.035	4	129	20	0.04	8.2	0.65	0.77	571	129	13.3	6.11
TZ-21	1.33	Taizi-lower	Black shale	4.20	0.455	0.044	2	119	56	0.06	6.8	0.34	0.62	418	119	24.1	3.51
TZ-23	1.33	Taizi-lower	Black shale	5.46				118	59	0.09	7.1	0.46	0.75	508	118	16.3	4.67
TZ-23-1	1.33	Taizi-lower	Black shale		0.785	0.061	4						0.75				
TZ-24	1.33	Taizi-lower	Black shale	3.71	0.452	0.044	2	208	17	0.21	9.1	0.60	1.94	139	208	7.0	4.59
TZ-25	1.33	Taizi-lower	Black shale	6.15	0.662	0.046	2	301	116	0.08	8.3	1.04	1.27	697	301	27.2	11.1
TZ-26	1.33	Taizi-lower	Black shale	6.56	0.652	0.056	3	241	98	0.43	7.8	0.48	0.85	913	241	31.0	7.19
TZ-27	1.33	Taizi-lower	Black shale	8.05	0.654	0.057	5	310	101	0.05	8.1	0.59	0.88	386	310	14.5	7.17

TZ-28	1.33	Taizi-lower	Black shale	4.84	0.660	0.030	5	174	20	0.05	6.6	0.40	0.60	130	174	5.1	4.36
DYP-1	1.40	Dayanping-upper	Black shale	0.74				93.1	5	9.19	10.2	0.88	10.15	185	93	1.8	2.35
DYP-2	1.40	Dayanping-upper	Black shale	0.56				76.2	5	12.82	8.7	0.75	8.72	157	76	1.5	1.53
DYP-3	1.40	Dayanping-upper	Black shale	0.33				88.3	<2	11.72	8.9	0.81	8.88	179	88	0.7	1.99
DYP-4	1.40	Dayanping-upper	Black shale	0.43	0.150	0.052	2	66.6	<2	15.92	6.2	0.62	6.23	134	67	0.6	1.68
DYP-5	1.40	Dayanping-upper	Black shale	0.56	0.077	0.048	2	93.1	<2	10.40	9.5	0.95	9.45	200	93	1.2	1.98
DYP-6	1.40	Dayanping-upper	Black shale	0.50				95.5	<2	11.35	8.6	0.88	8.57	170	96	0.5	1.96
DYP-7	1.40	Dayanping-upper	Black shale	0.55				78.6	<2	12.79	7.8	0.74	7.78	155	79	0.7	1.94
DYP2-1	1.40	Dayanping-lower	Black shale	0.70				79.3	<2	8.14	8.0	0.62	7.98	113	79	0.7	1.39
DYP2-2	1.40	Dayanping-lower	Black shale	0.49				75.8	4	13.66	7.2	0.55	7.18	116	76	1.2	1.44
DYP2-3	1.40	Dayanping-lower	Black shale	0.53				53	2	14.20	4.8	0.33	4.76	65	53	0.7	0.79
DYP2-4	1.40	Dayanping-lower	Black shale	0.54				101	<2	8.78	10.3	0.84	10.25	153	101	0.5	1.47
DYP2-5	1.40	Dayanping-lower	Black shale	0.87				90	<2	8.09	8.9	0.70	8.91	127	90	0.9	1.55
DYP2-6	1.40	Dayanping-lower	Black shale	0.81				96.4	<2	9.51	9.4	0.75	9.37	124	96	0.6	1.58
DYP2-7	1.40	Dayanping-lower	Black shale	0.68				98.2	<2	9.08	10.1	0.93	10.06	155	98	0.7	1.73
DYP2-8	1.40	Dayanping-lower	Black shale	0.85				101	<2	8.93	10.1	0.97	10.05	144	101	1.8	2.18
DYP2-9	1.40	Dayanping-lower	Black shale	0.72				79.7	<2	9.59	8.7	0.65	8.69	143	80	0.6	1.78
DYP2-10	1.40	Dayanping-lower	Black shale	0.51				89.6	<2	10.77	8.8	0.75	8.84	140	90	0.8	1.54
DYP2-11	1.40	Dayanping-lower	Black shale	0.72				88	3	9.87	8.9	0.73	8.85	152	88	0.8	1.83

DYP2-13	1.40	Dayanping-lower	Black shale	0.68				107	<2	9.01	10.6	1.04	10.57	161	107	0.8	2.04
DYP2-14	1.40	Dayanping-lower	Black shale	0.55				87.1	<2	9.30	9.0	0.73	8.99	142	87	0.5	1.49
DYP2-15	1.40	Dayanping-lower	Black shale	0.63	0.065	0.044	2	76.7	<2	12.01	7.7	0.65	7.68	145	77	0.5	1.72
DYP2-16	1.40	Dayanping-lower	Black shale	0.89	0.116	0.058	2	82.2	3	9.11	8.8	0.70	8.77	125	82	0.6	1.54
DYP2-17	1.40	Dayanping-lower	Black shale	0.62				99.1	<2	8.63	10.9	0.94	10.92	144	99	0.5	1.98
DYP2-18	1.40	Dayanping-lower	Black shale	0.70				94.9	<2	8.90	9.9	0.82	9.86	147	95	0.6	1.76
DYP2-19	1.40	Dayanping-lower	Black shale	0.75				85.9	<2	9.25	8.7	0.71	8.72	138	86	0.8	1.65

Supplementary Table 2. Rare Earth Elements plus Y for samples from the Shennongjia Group.

	La	Ce	Pr	Nd	Sm	Eu	Gd	Tb	Dy	Y	Ho	Er	Tm	Yb	Lu
Sample	μg/g	μg/g	μg/g	μg/g	μg/g	μg/g	μg/g	μg/g	μg/g	μg/g	μg/g	μg/g	μg/g	μg/g	μg/g
SZY-1	14.9	20.6	2.92	11.7	2.65	0.668	2.43	0.56	3.89	23.8	0.841	2.51	0.51	3.45	0.547
SZY-2	12.7	22	5.49	32.4	16.8	3.43	13.8	2.93	16.5	93.5	2.88	6.49	0.983	5.74	0.798
SZY-3	11	13.3	1.96	6.51	0.942	0.235	1.08	0.193	1.27	9.71	0.31	0.995	0.211	1.49	0.254
SZY-4	13.3	16.3	2.55	9.69	1.34	0.303	1.39	0.239	1.66	14.5	0.424	1.42	0.286	1.94	0.307
SZY-5	22.3	30.1	5.29	22.2	4.36	0.858	3.82	0.757	4.64	35.3	1.05	3.05	0.569	3.61	0.56
SZY-6	17.7	25.2	3.9	15.7	2.95	0.597	2.46	0.44	2.81	20.2	0.642	1.97	0.416	2.83	0.461
WSH-1	47.1	97.1	12.4	52.9	10.5	1.96	8.28	1.4	6.7	29.8	1.15	3.06	0.499	3.22	0.468
WSH-2	44.5	90.6	11.5	47.7	9.13	1.6	6.93	1.16	5.81	27.6	1.03	2.81	0.458	2.98	0.428
WSH-3	46.7	90.2	11.1	44.1	7.63	1.39	6.19	1.05	5.49	32.7	1.05	3.11	0.568	4.05	0.648
WSH-4	45.9	92.9	11.6	49.3	9.93	1.92	7.62	1.33	7.08	35.7	1.26	3.48	0.58	4.11	0.63
TZ1-1	173	86.3	28.4	119	15.4	2.32	12.9	1.82	7.61	49.3	1.41	4.13	0.51	3.02	0.426
TZ1-2	110	45.4	14.7	62.6	9.26	1.67	8.47	1.37	7.45	61.3	1.58	4.35	0.65	3.98	0.578
TZ1-3	71.6	31.8	9.08	40.1	6.48	1.31	6.06	0.979	5.02	41	0.996	2.56	0.353	1.94	0.269
TZ1-4	79	32.1	9.66	42.9	7.27	1.44	7.07	1.32	8.65	91.4	1.98	5.69	0.964	6.05	0.877
TZ1-5	112	64.6	20.2	86.2	11.7	1.94	9.84	1.49	6.63	44.3	1.3	3.69	0.488	3.03	0.423
TZ1-6	26.3	53.8	6.17	23.5	3.76	0.604	3.24	0.569	3.58	22.3	0.811	2.51	0.523	3.3	0.52
TZ1-7	33.8	65.4	7.49	28.2	4.33	0.787	3.77	0.651	4.04	24.9	0.897	2.81	0.593	3.8	0.556
TZ-1	79.4	131	14.9	53.5	8.65	1.39	7.75	1.16	5.71	26.8	1.07	3.05	0.555	3.4	0.463
TZ-2	121	215	23.8	85.5	13.4	1.95	11.6	1.75	8.49	41.4	1.61	4.75	0.827	5.44	0.79
TZ-3	15.7	28.2	3.41	13.1	2.93	0.673	2.88	0.775	5.73	32.7	1.17	3.12	0.638	3.96	0.557
TZ-4	106	188	22.2	83.5	15.7	2.34	15.6	2.76	16.4	122	3.61	9.65	1.7	9.82	1.46
TZ-5	107	187	20	68.4	10.3	1.32	9.49	1.42	6.86	35.8	1.24	3.53	0.597	3.8	0.533

TZ-6	18.2	40.4	5.88	26.4	6.04	1.18	4.75	1.04	6.55	34.3	1.36	3.71	0.749	4.79	0.686
TZ-7	69.9	127	17	68.4	10.8	1.54	7.74	1.17	5.78	33.2	1.17	3.74	0.651	4.36	0.622
TZ-8	157	268	31.3	114	14.8	2.01	13.8	1.83	7.7	43.1	1.46	4.3	0.722	4.54	0.646
TZ-9	50.4	89.2	10.8	39.4	6.52	1.26	5.7	0.966	5.11	26.1	1.02	2.83	0.545	3.53	0.491
TZ-10	68.3	111	12.9	45.8	7.16	1.2	6.53	1.04	5.46	31.3	1.12	3.33	0.641	4.21	0.6
TZ-11	84.8	156	18.3	66.7	10.8	1.6	9.16	1.34	6.3	31.7	1.18	3.4	0.581	3.89	0.556
TZ-13	46.5	94.8	14.3	64.6	11.8	1.44	6.83	1.13	5.65	32	1.18	3.62	0.669	4.21	0.607
TZ-14	89.8	139	16.4	56.8	8.55	1.28	7.7	1.12	5.63	31.6	1.11	3.23	0.569	3.64	0.51
TZ14-1	122	212	26.2	95.8	13.2	1.77	12.1	1.66	7.06	40	1.37	4.28	0.703	4.54	0.66
TZ-15	49.8	85.6	11.6	45.3	6.55	1.08	5.25	0.776	3.83	22.6	0.806	2.63	0.463	2.98	0.432
TZ-16	100	166	21.5	82.5	10.5	1.81	9.37	1.15	4.45	25.9	0.888	3.13	0.543	3.55	0.523
TZ16-1	4.54	7.56	0.896	3.01	0.415	0.091	0.505	0.087	0.504	3.24	0.109	0.32	0.066	0.394	0.058
TZ-17	28.1	55.6	8.87	38.8	6.62	1.42	4.87	0.883	5.27	36.4	1.22	3.71	0.742	4.7	0.704
TZ-18	33.6	52.4	7.52	28.2	4.73	1.02	4.08	0.652	3.47	23.4	0.789	2.37	0.459	3.04	0.444
TZ-20	28.1	37.4	5.41	19.8	2.93	0.607	2.83	0.436	2.86	22.5	0.691	2.25	0.498	3.42	0.556
TZ-21	24	33.3	5.19	19.2	2.9	0.555	2.68	0.444	2.83	20.7	0.645	1.97	0.405	2.6	0.41
TZ-23	27.6	37.6	6.06	22.6	3.35	0.628	3.17	0.54	3.4	25.8	0.817	2.5	0.502	3.36	0.504
TZ-24	41.4	48.8	8.81	32.7	5.19	0.947	4.55	0.726	3.89	28	0.896	2.88	0.581	4.36	0.718
TZ-25	24.5	29.6	5.5	20.5	3.22	0.642	3.3	0.635	4.43	35.3	1.1	3.3	0.696	4.8	0.777
TZ-26	36.2	51.6	8.75	35.6	7.05	1.26	6.01	1.14	6.51	39.8	1.38	3.94	0.767	5.38	0.865
TZ-27	41.6	55.8	9.02	35.3	5.67	0.941	4.97	0.72	4.06	29.6	0.976	3.1	0.64	4.49	0.728
TZ-28	32.1	45.6	6.77	24.9	3.78	0.579	3.71	0.601	3.93	31.8	0.985	3.4	0.736	5.27	0.877
TZ-28	32.1	45.6	6.77	24.9	3.78	0.579	3.71	0.601	3.93	31.8	0.985	3.4	0.736	5.27	0.877
DYP-1	37.9	66.9	8.61	34.5	6.43	1.49	5.84	1.05	5.49	33.3	1.05	2.93	0.554	3.34	0.478
DYP-2	28.5	50.3	6.66	27.3	5.28	1.16	4.51	0.835	4.45	26.5	0.871	2.34	0.422	2.54	0.361
DYP-3	34.5	62.7	8	32.2	6.02	1.28	5.31	0.954	5.21	31.1	1	2.72	0.46	2.82	0.373

DYP-4	21.7	38.1	4.98	20	3.78	0.912	3.51	0.652	3.79	24.2	0.744	1.97	0.355	2.11	0.292
DYP-5	32.6	58.3	7.53	30.1	5.85	1.25	5.1	0.923	5.1	31	1.02	2.72	0.491	2.9	0.417
DYP-6	31.5	55.4	7.05	28.3	5.13	1.14	4.7	0.831	4.65	28	0.928	2.5	0.425	2.64	0.38
DYP-7	27.4	48.1	6.17	24.8	4.55	1.01	4.04	0.76	4.28	27.8	0.867	2.38	0.424	2.61	0.36
DYP2-1	23.9	44.3	5.69	23.5	4.89	0.992	4.18	0.762	4.37	27.3	0.885	2.37	0.425	2.66	0.368
DYP2-2	25.3	46.4	5.83	24.5	4.84	1.23	4.2	0.819	4.59	30.4	0.921	2.4	0.429	2.61	0.369
DYP2-3	14.5	26.5	3.48	14.4	3.09	0.727	2.63	0.538	3.39	22.7	0.68	1.83	0.317	1.96	0.283
DYP2-4	32.2	59	7.41	29.5	5.5	1.18	4.93	0.892	4.83	29.5	0.972	2.58	0.467	2.89	0.386
DYP2-5	27	50.5	6.35	26	5.28	1.1	4.65	0.869	5.18	31	1.01	2.66	0.507	2.99	0.419
DYP2-6	30.7	55.4	6.82	27.1	5.15	1	4.57	0.839	4.89	28.7	0.969	2.53	0.484	2.89	0.395
DYP2-7	28.6	53	6.61	27.1	5.42	1.06	4.72	0.894	5.03	29.9	1.01	2.59	0.468	2.75	0.399
DYP2-8	35.8	65.5	8.19	33	6.22	1.18	5.45	0.987	5.4	32	1.09	2.88	0.52	3.12	0.436
DYP2-9	23.1	43.8	5.55	23.2	4.96	0.975	4.38	0.857	5.08	30.4	1.02	2.73	0.491	3	0.438
DYP2-10	29.7	55.7	7.17	29.2	5.89	1.21	5.15	0.932	5.18	31.9	1.04	2.67	0.488	2.86	0.398
DYP2-11	27.5	51.2	6.46	26.1	5.11	1.03	4.41	0.826	4.6	27.8	0.92	2.43	0.429	2.7	0.396
DYP2-13	39.6	73.5	9.04	35.9	6.44	1.26	5.73	1.03	5.61	34.7	1.14	3.1	0.564	3.2	0.483
DYP2-14	26.2	48.5	6.17	25.1	4.94	1.01	4.3	0.83	4.81	27.6	0.915	2.48	0.434	2.67	0.391
DYP2-15	26.8	48.4	6.25	25	4.8	0.985	4.31	0.802	4.51	28.7	0.913	2.47	0.455	2.6	0.383
DYP2-16	28.5	51.9	6.46	26.4	5.09	1.05	4.59	0.832	4.82	29.3	0.945	2.58	0.459	2.78	0.392
DYP2-17	36.7	67.4	8.35	33	6.06	1.07	5.33	0.972	5.39	31.1	1.08	2.91	0.524	3.22	0.459
DYP2-18	30.6	56.5	7.02	28.5	5.65	1.12	4.81	0.89	5.08	31.1	1.02	2.76	0.51	3.09	0.439
DYP2-19	25.9	48.2	6.03	24.8	5.02	0.984	4.46	0.837	4.83	29.3	0.977	2.66	0.482	2.88	0.424

Supplementary Table 3. Summary of sediment chromium isotope data from 2000 Ma to present.

Formation/Sample name	Age (Ma)	rock type	$\delta^{53}Cr$	2 SE	Cr/Al	Cr/Ti	ref
			per mil	per mil	ppm/wt %	g/g	
Zhengjiaya	1083	Black shale	0.039	0.047	13.4	0.029	present study
Zhengjiaya	1083	Black shale	0.288	0.044	33.0	0.062	present study
Zhengjiaya	1083	Black shale	0.270	0.048	33.9	0.065	present study
Zhengjiaya	1083	Black shale	0.229	0.045	28.1	0.056	present study
Zhengjiaya	1083	Black shale	0.364	0.047	25.3	0.049	present study
Zhengjiaya	1083	Black shale	0.086	0.043	16.7	0.036	present study
Wenshuihe	1180	Black shale	0.181	0.046	26.6	0.011	present study
Wenshuihe	1180	Black shale	0.197	0.042	30.7	0.013	present study
Wenshuihe	1180	Black shale	0.285	0.116	38.1	0.017	present study
Wenshuihe	1180	Black shale	0.091	0.054	32.5	0.017	present study
Taizi-upper	1330	Black shale	0.691	0.052	44.9	0.058	present study
Taizi-upper	1330	Black argillaceous limestone	0.763	0.047	45.8	0.044	present study
Taizi-upper	1330	Black argillaceous limestone	0.868	0.044	24.5	0.006	present study
Taizi-upper	1330	Black argillaceous limestone	0.798	0.049	49.3	0.045	present study
Taizi-upper	1330	Black limey mudstone	0.789	0.047	36.5	0.039	present study
Taizi-upper	1330	Black shale	0.359	0.061	23.5	0.026	present study
Taizi-lower	1330	Black-gray shale	-0.269	0.059	10.5	0.017	present study
Taizi-lower	1330	Black shale	-0.280	0.050	9.6	0.014	present study
Taizi-lower	1330	Black shale	-0.216	0.051	8.5	0.009	present study
Taizi-lower	1330	Black shale	0.107	0.044	10.1	0.011	present study
Taizi-lower	1330	chert	-0.025	0.061	383.0	0.457	present study
Taizi-lower	1330	Black sandstone	0.072	0.025	9.3	0.009	present study
Taizi-lower	1330	Black shale	0.197	0.038	11.7	0.015	present study
Taizi-lower	1330	Black shale	0.496	0.035	29.7	0.033	present study
Taizi-lower	1330	Black shale	0.455	0.044	32.9	0.058	present study

Taizi-lower	1330	Black shale	0.785	0.061		0.043	present study
Taizi-lower	1330	Black shale	0.452	0.044	43.3	0.059	present study
Taizi-lower	1330	Black shale	0.662	0.046	68.4	0.049	present study
Taizi-lower	1330	Black shale	0.652	0.056	58.8	0.084	present study
Taizi-lower	1330	Black shale	0.654	0.057	72.2	0.088	present study
Taizi-lower	1330	Black shale	0.660	0.030	49.9	0.073	present study
Dayanping-upper	1400	Black shale	-0.150	0.052	20.2	0.018	present study
Dayanping-upper	1400	Black shale	-0.077	0.048	18.6	0.017	present study
Dayanping-lower	1400	Black shale	-0.065	0.044	18.9	0.020	present study
Dayanping-lower	1400	Black shale	-0.116	0.058	17.7	0.020	present study
Linok	970	carbonate	-0.23	0.04	4.8		[3]
Sukh. Tung.	970	carbonate	-0.22	0.03	6.5		[3]
Sukh. Tung.	970	carbonate	-0.11	0.03	5.1		[3]
Sukh. Tung.	970	carbonate	0.08	0.06	7.9		[3]
Sukh. Tung.	970	carbonate	0.37	0.05	29.2		[3]
Burovaya	970	carbonate	-0.23	0.05	5.1		[3]
Miroyedikha	970	carbonate	-0.09	0.02	4.9		[3]
Miroyedikha	970	carbonate	0.69	0.05	7.0		[3]
Miroyedikha	970	carbonate	0	0.03	4.8		[3]
Miroyedikha	970	carbonate	0.5	0.04	7.7		[3]
Miroyedikha	970	carbonate	0.93	0.03	7.3		[3]
Miroyedikha	970	carbonate	0.6	0.04	6.6		[3]
Turukhansk	970	carbonate	1.28	0.02	5.7		[3]
Turukhansk	970	carbonate	0.6	0.05	5.9		[3]
Turukhansk	970	carbonate	0.7	0.03	7.7		[3]
Turukhansk	970	carbonate	1.43	0.05	18.7		[3]
Turukhansk	970	carbonate	0.58	0.03	9.1		[3]
Turukhansk	970	carbonate	0.78	0.05	7.5		[3]
Turukhansk	970	carbonate	-0.04	0.04	6.2		[3]
Miroyedikha	970	carbonate	0.4	0.02	6.9		[3]
Miroyedikha	970	carbonate	0.07	0.07	4.3		[3]

Ser. do P. Ver.	1112	carbonate	0.55	0.08	3.5	3
Mor. do Calc.	1112	carbonate	1.27	0.09	29.9	3
Mor. do Calc.	1112	carbonate	0.7	0.09	3.3	3
Mor. do Calc.	1112	carbonate	0.18	0.08	2.3	3
Mor. do Calc.	1112	carbonate	0.49	0.07	8.0	3
Lapa	1112	carbonate	0.31	0.08	16.4	3
Lapa	1112	carbonate	0.27	0.08	7.2	3
Lapa	1112	carbonate	0.22	0.07	13.0	3
Lapa	1112	carbonate	0.68	0.05	3.2	3
Lapa	1112	carbonate	-0.07	0.06	1.9	3
Lapa	1112	carbonate	-0.05	0.06	2.3	3
Lapa	1112	carbonate	-0.11	0.07	0.5	3
Lapa	1112	carbonate	-0.15	0.04	1.4	3
Lapa	1112	carbonate	-0.19	0.08	6.7	3
Lapa	1112	carbonate	-0.17	0.05	5.6	3
Lapa	1112	carbonate	-0.14	0.05	8.5	3
Lapa	1112	carbonate	-0.23	0.07	7.3	3
Lapa	1112	carbonate	-0.15	0.08	1.6	3
Lapa	1112	carbonate	-0.11	0.08	1.5	3
Lapa	1112	carbonate	-0.16	0.06	3.1	3
Lapa	1112	carbonate	-0.15	0.06	4.5	3
Lapa	1112	carbonate	-0.08	0.06	0.7	3
Lapa	1112	carbonate	-0.19	0.06	4.0	3
Lapa	1112	carbonate	-0.12	0.06	0.6	3
Lapa	1112	carbonate	-0.13	0.05	7.2	3
Lapa	1112	carbonate	-0.21	0.06	2.2	3
Lapa	1112	carbonate	-0.09	0.06	3.7	3
Lapa	1112	carbonate	0.05	0.07	3.9	3
Lapa	1112	carbonate	-0.12	0.06	4.3	3
Lapa	1112	carbonate	-0.13	0.08	2.0	3
Mor. do Calc.	1112	carbonate	0.6	0.08	6.0	3
Lapa	1112	carbonate	-0.1	0.07	0.7	3

Lapa	1112	carbonate	-0.11	0.07	1.4	3
Lapa	1112	carbonate	-0.06	0.09	1.5	3
Lapa	1112	carbonate	-0.21	0.07	1.4	3
Lapa	1112	carbonate	-0.13	0.08	0.5	3
Lapa	1112	carbonate	-0.04	0.06	5.6	3
Lapa	1112	carbonate	-0.18	0.05	0.8	3
Lapa	1112	carbonate	-0.12	0.08	3.3	3
Lapa	1112	carbonate	-0.15	0.07	1.4	3
Lapa	1112	carbonate	-0.04	0.06	1.5	3
Lapa	1112	carbonate	-0.09	0.08	2.4	3
Lapa	1112	carbonate	-0.12	0.07	3.6	3
Touirist	1107	carbonate	0.39	0.01	6.8	3
Ag. el Mabha	1107	carbonate	0.04	0.09	1.3	3
Ag. el Mabha	1107	carbonate	-0.13	0.06	3.1	3
Ag. el Mabha	1107	carbonate	-0.02	0.04	1.3	3
Ag. el Mabha	1107	carbonate	0.03	0.06	1.6	3
Ag. el Mabha	1107	carbonate	-0.16	0	1.2	3
Ag. el Mabha	1107	carbonate	-0.05	0.03	1.4	3
Ag. el Mabha	1107	carbonate	-0.1	0.05	1.5	3
Gouamir	1107	carbonate	-0.1	0.05	2.7	3
Gouamir	1107	carbonate	-0.24	0.03	2.0	3
Gouamir	1107	carbonate	0.03	0.02	3.1	3
Gouamir	1107	carbonate	-0.12	0.02	1.9	3
Gouamir	1107	carbonate	-0.29	0.04	3.5	3
Gouamir	1107	carbonate	-0.2	0.04	2.5	3
Gouamir	1107	carbonate	-0.11	0.04	2.4	3
Gouamir	1107	carbonate	-0.09	0.04	3.2	3
En Nesoar	1107	carbonate	0.28	0.04	11.1	3
En Nesoar	1107	carbonate	0.68	0.03	27.6	3
Angmaat	1092	carbonate	0.15	0.02	7.3	3
Angmaat	1092	carbonate	0.07	0.05	7.7	3
Angmaat	1092	carbonate	0.09	0.09	3.9	3

Angmaat	1092	carbonate	0.16	0.02	4.5		3
Angmaat	1092	carbonate	0.07	0.03	4.0		3
Angmaat	1092	carbonate	0.04	-	12.4		3
Angmaat	1092	carbonate	0.41	0.03	11.9		3
Angmaat	1092	carbonate	0.5	0.03	10.7		3
Angmaat	1092	carbonate	1.77	0.03	15.9		3
Angmaat	1092	carbonate	0.12	0.03	8.8		3
Angmaat	1092	carbonate	-0.13	0.03	4.1		3
Angmaat	1092	carbonate	-0.16	-	6.8		3
Angmaat	1092	carbonate	0.04	0.03	6.4		3
Angmaat	1092	carbonate	0.19	0.06	4.9		3
Angmaat	1092	carbonate	0.73	0.12	4.7		3
Angmaat	1092	carbonate	-0.04	0.02	14.8		3
Angmaat	1092	carbonate	0.83	0.01	-		3
Angmaat	1092	carbonate	0.44	0.02	6.9		3
Angmaat	1092	carbonate	-0.04	0.06	5.5		3
Doldgeville Fm	450	shale	0.28	0.05		0.028	4
Doldgeville Fm	450	shale	0.34	0.04		0.030	4
Doldgeville Fm	450	shale	0.58	0.05		0.020	4
Doldgeville Fm	450	shale	0.49	0.05		0.022	4
Tal Group	550	shale	0.23	0.04		0.031	4
Tal Group	550	shale	0.46	0.05		0.048	4
Tal Group	550	shale	0.55	0.05		0.047	4
Tal Group	550	shale	0.59	0.04		0.047	4
Tal Group	550	shale	0.16	0.03		0.066	4
Tal Group	550	shale	0.53	0.04		0.067	4
Tal Group	550	shale	0.17	0.05		0.033	4
Doushantou	630	shale	-0.13	0.04		0.014	4
Doushantou	630	shale	-0.11	0.03		0.015	4
Doushantou	630	shale	-0.16	0.03		0.014	4
Doushantou	630	shale	-0.04	0.03		0.015	4

Doushantou	630	shale	-0.05	0.03	0.014	4
Doushantou	630	shale	-0.1	0.03	0.013	4
Tongari Group	640	shale	-0.16	0.03	0.020	4
Tongari Group	640	shale	0.1	0.03	0.018	4
Tongari Group	640	shale	0.14	0.03	0.016	4
Tongari Group	640	shale	0.16	0.04	0.016	4
Tongari Group	640	shale	0.12	0.03	0.012	4
Tongari Group	800	shale	0.09	0.03	0.089	4
Tongari Group	800	shale	0.23	0.04	0.107	4
Tongari Group	800	shale	0.24	0.05	0.113	4
Chuar	740	shale	0.7	0.05	0.033	4
Chuar	740	shale	0.73	0.05	0.034	4
Chuar	740	shale	0.68	0.05	0.033	4
Chuar	740	shale	0.7	0.05	0.039	4
Chuar	740	shale	0.58	0.04	0.021	4
Chuar	740	shale	0.62	0.03	0.020	4
Chuar	740	shale	0.73	0.03	0.027	4
Chuar	740	shale	0.6	0.03	0.024	4
Chuar	740	shale	0.52	0.03	0.021	4
Chuar	740	shale	0.15	0.03	0.017	4
Simla	840	shale	-0.04	0.04	0.033	4
Simla	840	shale	0.04	0.04	0.028	4
Simla	840	shale	0.08	0.04	0.036	4
Simla	840	shale	0.1	0.05	0.050	4
Simla	840	shale	0.16	0.05	0.022	4
Simla	840	shale	0.09	0.05	0.046	4
Simla	840	shale	-0.09	0.03	0.024	4
Simla	840	shale	-0.08	0.03	0.018	4
Arctic Bay	1100	shale	-0.07	0.02	0.021	4
Arctic Bay	1100	shale	-0.12	0.02	0.022	4
Arctic Bay	1100	shale	-0.14	0.02	0.018	4
Arctic Bay	1100	shale	0.08	0.02	0.019	4

Arctic Bay	1100	shale	0.01	0.02	0.019	4
Arctic Bay	1100	shale	0.06	0.02	0.019	4
Arctic Bay	1100	shale	0.07	0.02	0.022	4
Arctic Bay	1100	shale	0.01	0.02	0.021	4
Arctic Bay	1100	shale	-0.07	0.02	0.022	4
Arctic Bay	1100	shale	-0.1	0.02	0.024	4
Arctic Bay	1100	shale	-0.1	0.02	0.024	4
Arctic Bay	1100	shale	-0.13	0.02	0.020	4
Arctic Bay	1100	shale	-0.13	0.02	0.020	4
Arctic Bay	1100	shale	-0.12	0.02	0.017	4
Arctic Bay	1100	shale	-0.05	0.02	0.019	4
Arctic Bay	1100	shale	-0.09	0.02	0.019	4
Arctic Bay	1100	shale	-0.04	0.01	0.021	4
Arctic Bay	1100	shale	-0.03	0.02	0.022	4
Arctic Bay	1100	shale	0.09	0.02	0.020	4
Arctic Bay	1100	shale	-0.11	0.02	0.023	4
Arctic Bay	1100	shale	-0.03	0.02	0.022	4
Arctic Bay	1100	shale	-0.09	0.02	0.022	4
Arctic Bay	1100	shale	-0.1	0.02	0.022	4
Arctic Bay	1100	shale	-0.13	0.01	0.018	4
Arctic Bay	1100	shale	0.03	0.02	0.022	4
Arctic Bay	1100	shale	-0.09	0.02	0.021	4
Arctic Bay	1100	shale	-0.16	0.02	0.020	4
Xiamaling	1380	shale	-0.05	0.03	0.014	4
Xiamaling	1380	shale	-0.03	0.02	0.014	4
Xiamaling	1380	shale	-0.09	0.02	0.017	4
Xiamaling	1380	shale	-0.04	0.02	0.016	4
Xiamaling	1380	shale	0.17	0.02	0.015	4
Xiamaling	1380	shale	-0.19	0.03	0.014	4
Velkerri	1400	shale	-0.14	0.02	0.017	4
Velkerri	1400	shale	-0.01	0.02	0.017	4
Velkerri	1400	shale	0.05	0.02	0.018	4

Velkerri	1400	shale	-0.13	0.02	0.012	[4]
Velkerri	1400	shale	-0.09	0.02	0.013	[4]
Velkerri	1400	shale	-0.14	0.03	0.012	[4]
Velkerri	1400	shale	-0.14	0.02	0.013	[4]
Velkerri	1400	shale	-0.13	0.02	0.013	[4]
Barney Creek	1650	shale	-0.17	0.02	0.014	[4]
Barney Creek	1650	shale	-0.34	0.02	0.016	[4]
Barney Creek	1650	shale	-0.11	0.03	0.016	[4]
Barney Creek	1650	shale	-0.14	0.02	0.024	[4]
Barney Creek	1650	shale	-0.13	0.02	0.017	[4]
Barney Creek	1650	shale	-0.09	0.02	0.015	[4]
Barney Creek	1650	shale	-0.14	0.02	0.012	[4]
Barney Creek	1650	shale	-0.14	0.02	0.013	[4]
Barney Creek	1650	shale	-0.11	0.02	0.017	[4]
Barney Creek	1650	shale	-0.14	0.02	0.028	[4]
Mt. Les	1650	shale	-0.1	0.02	0.016	[4]
Mt. Les	1650	shale	-0.17	0.02	0.015	[4]
Mt. Les	1650	shale	-0.28	0.03	0.009	[4]
Mt. Les	1650	shale	-0.08	0.03	0.016	[4]
Lady Lorreta	1650	shale	-0.18	0.02	0.018	[4]
Lady Lorreta	1650	shale	-0.19	0.02	0.018	[4]
Lady Lorreta	1650	shale	-0.18	0.01	0.042	[4]
Lady Lorreta	1650	shale	-0.2	0.02	0.019	[4]
Lady Lorreta	1650	shale	-0.17	0.03	0.021	[4]
Lady Lorreta	1650	shale	-0.23	0.03	0.015	[4]
Lady Lorreta	1650	shale	-0.2	0.02	0.017	[4]
Damtha	1700	shale	-0.15	0.02	0.016	[4]
Damtha	1700	shale	-0.06	0.02	0.016	[4]
Damtha	1700	shale	-0.09	0.02	0.017	[4]
Damtha	1700	shale	-0.14	0.03	0.017	[4]
Damtha	1700	shale	-0.13	0.02	0.017	[4]

PPeru margin	0	clastic sediment	0.57	0.059	5
PPeru margin	0	clastic sediment	0.59	0.058	5
Peru margin	0	clastic sediment	0.57	0.067	5
Peru margin	0	clastic sediment	0.55	0.064	5
Peru margin	0	clastic sediment	0.63	0.115	5
Peru margin	0	clastic sediment	0.64	0.070	5
Peru margin	0	clastic sediment	0.6	0.069	5
Peru margin	0	clastic sediment	0.59	0.070	5
Peru margin	0	clastic sediment	0.56	0.057	5
Peru margin	0	clastic sediment	0.65	0.030	5
Peru margin	0	clastic sediment	0.72	0.019	5
Peru margin	0	clastic sediment	0.73	0.055	5
Peru margin	0	clastic sediment	0.73	0.029	5
Peru margin	0	clastic sediment	0.64	0.025	5
Peru margin	0	clastic sediment	0.57	0.019	5
Peru margin	0	clastic sediment	0.47	0.057	5
Peru margin	0	clastic sediment	0.42	0.037	5
Peru margin	0	clastic sediment	0.55	0.046	5
Peru margin	0	clastic sediment	0.75	0.090	5
Peru margin	0	clastic sediment	0.8	0.086	5
Peru margin	0	clastic sediment	0.75	0.087	5
Peru margin	0	clastic sediment	0.88	0.081	5
Peru margin	0	clastic sediment	0.83	0.065	5
Peru margin	0	clastic sediment	0.67	0.049	5
Peru margin	0	clastic sediment	0.74	0.053	5
Peru margin	0	clastic sediment	0.91	0.059	5
Peru margin	0	clastic sediment	0.75	0.070	5
Peru margin	0	clastic sediment	0.82	0.041	5
Peru margin	0	clastic sediment	0.75	0.032	5
Peru margin	0	clastic sediment	0.62	0.088	5
Peru margin	0	clastic sediment	0.5	0.052	5
Peru margin	0	clastic sediment	0.47	0.045	5

Peru margin	0	clastic sediment	0.44	0.037	[5]
Cariaco Basin	0	clastic sediment	0.31	0.036	[6]
Cariaco Basin	0	clastic sediment	0.25	0.033	[6]
Cariaco Basin	0	clastic sediment	0.28	0.035	[6]
Cariaco Basin	0	clastic sediment	0.17	0.033	[6]
Cariaco Basin	0	clastic sediment	0.29	0.034	[6]
Cariaco Basin	0	clastic sediment	0.29	0.034	[6]
Cariaco Basin	0	clastic sediment	0.17	0.034	[6]
Oxic Marine	0	clastic sediment	-0.06	0.017	[5]
Oxic Marine	0	clastic sediment	0.06	0.018	[5]
Oxic Marine	0	clastic sediment	-0.06	0.012	[5]
Oxic Marine	0	clastic sediment	-0.06	0.012	[5]
Oxic Marine	0	clastic sediment	-0.01	0.024	[5]
Oxic Marine	0	clastic sediment	-0.05	0.019	[5]
Oxic Marine	0	clastic sediment	-0.07	0.017	[5]
Oxic Marine	0	clastic sediment	-0.08	0.016	[5]
Oxic Marine	0	clastic sediment	-0.08	0.015	[5]
Oxic Marine	0	clastic sediment	-0.13	0.017	[5]
Oxic Marine	0	clastic sediment	-0.14	0.002	[5]
Oxic Marine	0	clastic sediment	-0.13	0.016	[5]
Oxic Marine	0	clastic sediment	-0.08	0.011	[5]
Oxic Marine	0	clastic sediment	-0.06	0.014	[5]
Oxic Marine	0	clastic sediment	-0.02	0.023	[5]
Oxic Marine	0	clastic sediment	-0.03	0.017	[5]
Oxic Marine	0	clastic sediment	-0.03	0.017	[5]
		clastic sediment			
Oxic Marine	0	clastic sediment	-0.07	0.019	[5]
Oxic Marine	0	clastic sediment	0	0.022	[5]
Oxic Marine	0	clastic sediment	0.23	0.023	[5]

Oxic Marine	0	clastic sediment	0.02		0.015	5
Oxic Marine	0	clastic sediment	-0.01		0.013	5
Oxic Marine	0	clastic sediment	-0.1		0.039	5
Oxic Marine	0	clastic sediment	-0.11		0.012	5
Oxic Marine	0	clastic sediment	-0.1		0.013	5
Cerro Espuelitas Fm	550	iron formation	1.32	0.04		7
Cerro Espuelitas Fm	550	iron formation	0.64	0.09		7
Cerro Espuelitas Fm	550	iron formation	0.62	0.11		7
Cerro Espuelitas Fm	550	iron formation	0.24	0.09		7
Polanco Fm	550	iron formation	1.17	0.12		7
Yerbal Fm	550	iron formation	3.64	0.13		7
Yerbal Fm	550	iron formation	0.99	0.11		7
Yerbal Fm	550	iron formation	0.27	0.09		7
Yerbal Fm	550	iron formation	0.11	0.07		7
Yerbal Fm	550	iron formation	1.13	0.13		7
Yerbal Fm	550	iron formation	1.24	0.12		7
Yerbal Fm	550	iron formation	1.74	0.14		7
Yerbal Fm	550	iron formation	0.63	0.09		7
Yerbal Fm	550	shale	-0.04	0.09		7
Yerbal Fm	550	Siltstone	-0.15	0.09		7
Polanco Fm	550	Limestone	-0.21	0.08		7
Yerbal Fm	570	Limestone	-0.11	0.02		7
Yerbal Fm	570	iron formation	-0.21	0.01		7
Yerbal Fm	570	siltstone	-0.17	0.03		7
Yerbal Fm	570	siltstone	-0.24	0.07		7
Yerbal Fm	570	siltstone	-0.22	0.02		7
Yerbal Fm	570	siltstone	-0.16			7
Yerbal Fm	570	siltstone	-0.20			7
Yerbal Fm	570	siltstone	-0.11			7

Yerbal Fm	570	siltstone	-0.10	7
Yerbal Fm	570	siltstone	-0.24	7
Yerbal Fm	570	siltstone	-0.14	7
Yerbal Fm	570	siltstone	-0.23	7
Yerbal Fm	570	siltstone	-0.25	7
Yerbal Fm	570	siltstone	-0.24	7
Yerbal Fm	570	siltstone	-0.24	7
Yerbal Fm	570	siltstone	-0.09	7
Yerbal Fm	570	siltstone	-0.06	7
Yerbal Fm	570	siltstone	0.52	7
Yerbal Fm	570	siltstone	-0.06	7
Yerbal Fm	570	siltstone	0.09	7
Yerbal Fm	570	siltstone	-0.12	7
Yerbal Fm	570	siltstone	-0.22	7
Yerbal Fm	570	siltstone	-0.06	7
Yerbal Fm	570	siltstone	-0.19	7
Yerbal Fm	570	siltstone	-0.23	7
Yerbal Fm	570	siltstone	-0.17	7
Yerbal Fm	570	siltstone	-0.27	7
Yerbal Fm	570	siltstone	-0.13	7
Yerbal Fm	570	siltstone	0.10	7
Yerbal Fm	570	siltstone	0.12	7
Yerbal Fm	570	siltstone	-0.13	7
Yerbal Fm	570	siltstone	0.09	7
Yerbal Fm	570	Siltstone	-0.06	7
Yerbal Fm	570	Siltstone	-0.13	7
Yerbal Fm	570	Siltstone	-0.07	7
Yerbal Fm	570	Siltstone	-0.09	7
Yerbal Fm	570	Siltstone	-0.14	7
Yerbal Fm	570	Siltstone	-0.10	7
Yerbal Fm	570	Siltstone	0.60	7
Yerbal Fm	570	chert	0.67	7

Yerbal Fm	570	chert	1.05		7
Yerbal Fm	570	Dolostone	1.08		7
Yerbal Fm	570	Dolostone	1.18		7
Yerbal Fm	570	Dolostone	-0.10		7
Yerbal Fm	570	Dolostone	-0.11		7
Yerbal Fm	570	Siltstone	-0.17		7
CC-1A	550	iron formation	1.34	0.14	8
CC-1A	550	iron formation	1.32	0.04	8
CC-1B	550	iron formation	0.52	0.13	8
CC-1B	550	iron formation	0.44	0.11	8
SAL-4A	570	iron formation	3.22	0.03	8
SAL-4A	570	iron formation	0.04	0.03	8
SAL-4A	570	iron formation	0.03	0.04	8
SAL-4B	570	iron formation	4.92	0.02	8
SAL-4B	570	iron formation	5	0.02	8
SAL-4C	570	iron formation	4.51	0.03	8
SAL-4D	570	iron formation	3.12	0.18	8
SAL-4E	570	iron formation	2.09	0.07	8
SAL-4E	570	iron formation	0.08	0.05	8
SAL-4E	570	iron formation	0.1	0.05	8
SAL-4E	570	iron formation	0.06	0.05	8
SAL-4E	570	iron formation	0.09	0.03	8
SAL-4E	570	iron formation	2.03	0.04	8
SAL-4F	570	iron formation	0.31	0.03	8
SAL-5A	570	iron formation	0.98	0.05	8
SAL-5A	570	iron formation	1.1	0.05	8
SAL-5A	570	iron formation	1.1	0.02	8
SAL-5A	570	iron formation	0.96	0.02	8
SAL-5A	570	iron formation	0.85	0.07	8
SAL-5B	570	iron formation	0.29	0.03	8
SAL-5B	570	iron formation	0.04	0.05	8

SAL-5B	570	iron formation	0.27	0.02	8
SAL-5C	570	iron formation	5.4	0.65	8
SAL-5C	570	iron formation	0.04	0.05	8
SAL-5D	570	iron formation	1.06	0.03	8
SAL-5D	570	iron formation	1.19	0.03	8
SAL-5D	570	iron formation	1.21	0.17	8
CC-1A	550	iron formation	1.34	0.14	8
CC-1A	550	iron formation	1.32	0.04	8
CC-1B	550	iron formation	0.52	0.13	8
CC-1B	550	iron formation	0.44	0.11	8
Y-5	740	iron formation	0.9	0.03	8
Y-5	740	iron formation	0.96	0.04	8
Y-5	740	iron formation	0.05	0.03	8
JK-1	1874	iron formation	-0.25	0.06	8
JK-1	1874	iron formation	-0.14	0.05	8
R-3-89-1	1880	iron formation	-0.25	0.02	8
R-3-89-2	1880	iron formation	-0.17	0.03	8
R-3-89-3	1880	iron formation	-0.17	0.04	8
R-5	1840	iron formation	0.21	0.02	8
R-3	1840	iron formation	0.08	0.04	8
R-3	1840	iron formation	0.04	0.05	8
R-2	1840	iron formation	0.14	0.02	8
S-25	1840	iron formation	0.04	0.08	8
S-24	1840	iron formation	0.06	0.02	8
S-23	1840	iron formation	0.08	0.02	8
S-21	1874	iron formation	-0.19	0.02	8
S-21	1874	iron formation	-0.18	0.02	8
S-20	1874	iron formation	-0.13	0.04	8
S-20	1874	iron formation	-0.11	0.03	8
S-16	1874	iron formation	-0.23	0.02	8
S-16	1874	iron formation	-0.15	0.04	8
S-13	1874	iron formation	-0.18	0.02	8

S-13	1874	iron formation	-0.11	0.02		8
S-10	1874	iron formation	-0.24	0.04		8
S-8	1874	iron formation	-0.26	0.02		8
S-4	1874	iron formation	-0.22	0.05		8
S-2	1874	iron formation	-0.12	0.08		8
Langrial	62	oolitic ironstones	0.50	0.04	0.245	1
Langrial	62	oolitic ironstones	0.56	0.04	0.116	1
Langrial	62	oolitic ironstones	0.48	0.03	0.119	1
Langrial	62	oolitic ironstones	0.25	0.04	0.043	1
Langrial	62	oolitic ironstones	0.38	0.03	0.076	1
Langrial	62	oolitic ironstones	0.60	0.04	0.123	1
Langrial	62	oolitic ironstones	0.55	0.03	0.110	1
Rashby Ironstone	180	oolitic ironstones	0.57	0.03	0.150	1
Cleveland Ironstone	183	oolitic ironstones	0.11	0.04	0.092	1
Cleveland Ironstone	183	oolitic ironstones	-0.01	0.03	0.067	1
Scunthorpe	190	oolitic ironstones	0.62	0.03	0.186	1
Scunthorpe	190	oolitic ironstones	0.48	0.04	0.163	1
Scunthorpe	190	oolitic ironstones	0.37	0.03	0.056	1
Scunthorpe	190	oolitic ironstones	0.05	0.05	0.048	1
Cap de la Che`vre	460	oolitic ironstones	-0.08	0.02	0.012	1
Cap de la Che`vre	460	oolitic ironstones	-0.03	0.03	0.019	1
Cap de la Che`vre	460	oolitic ironstones	-0.09	0.02	0.018	1
Cap de la Che`vre	460	oolitic ironstones	0.11	0.05	0.025	1
Red Mountain	440	oolitic ironstones	0.15	0.02	0.029	1
Red Mountain	440	oolitic ironstones	0.48	0.02	0.138	1
Red Mountain	440	oolitic ironstones	0.08	0.02	0.021	1
Red Mountain	440	oolitic ironstones	0.39	0.02	0.056	1
Red Mountain	440	oolitic ironstones	0.65	0.02	0.147	1
Aok	900	oolitic ironstones	-0.08	0.02	0.597	1
Aok	900	oolitic ironstones	-0.10	0.03	0.550	1
Sherwin	1450	oolitic ironstones	-0.13	0.04	0.225	1

Sherwin	1450	oolitic ironstones	-0.19	0.03	0.088	1
Sherwin	1450	oolitic ironstones	-0.07	0.04	0.056	1
Sherwin	1450	oolitic ironstones	-0.01	0.03	0.051	1
Sherwin	1450	oolitic ironstones	-0.05	0.03	0.081	1
Sherwin	1450	oolitic ironstones	-0.02	0.02	0.190	1
Sherwin	1450	oolitic ironstones	-0.07	0.02	0.092	1
Sherwin	1450	oolitic ironstones	-0.05	0.03	0.186	1
Sherwin	1450	oolitic ironstones	-0.13	0.02	0.074	1
Sherwin	1450	oolitic ironstones	-0.10	0.03	0.147	1
Sherwin	1450	oolitic ironstones	-0.15	0.02	0.068	1
Sherwin	1450	oolitic ironstones	-0.02	0.02	0.113	1
Sherwin	1450	oolitic ironstones	-0.01	0.04	0.200	1
Sherwin	1450	oolitic ironstones	-0.03	0.02	0.057	1
Sherwin	1450	oolitic ironstones	-0.03	0.03	0.044	1
Freedom	1650	oolitic ironstones	-0.11	0.03	0.052	1
Freedom	1650	oolitic ironstones	-0.12	0.02	0.037	1
Freedom	1650	oolitic ironstones	-0.18	0.02	0.060	1
Freedom	1650	oolitic ironstones	-0.03	0.03	0.050	1
Freedom	1650	oolitic ironstones	-0.10	0.02	0.082	1
Freedom	1650	oolitic ironstones	-0.09	0.02	0.064	1
Chuanlinggou	1700	oolitic ironstones	-0.16	0.03	0.049	1
Chuanlinggou	1700	oolitic ironstones	-0.17	0.05	0.120	1
Chuanlinggou	1700	oolitic ironstones	-0.10	0.06	0.110	1
Chuanlinggou	1700	oolitic ironstones	-0.20	0.04	0.125	1
Chuanlinggou	1700	oolitic ironstones	-0.05	0.04	0.205	1
Chuanlinggou	1700	oolitic ironstones	-0.21	0.02	0.453	1
Chuanlinggou	1700	oolitic ironstones	-0.13	0.02	0.205	1
Chuanlinggou	1700	oolitic ironstones	-0.16	0.02	0.042	1
Chuanlinggou	1700	oolitic ironstones	-0.18	0.02	0.032	1
Chuanlinggou	1700	oolitic ironstones	0.03	0.03	0.185	1
Demerara Rise	94	shale	1.23	0.03	0.250	1

Demerara Rise	94	shale	0.86	0.03	0.110	1
Demerara Rise	94	shale	0.83	0.03	0.110	1
Demerara Rise	94	shale	1.05	0.04	0.140	1
Demerara Rise	94	shale	1.06	0.03	0.100	1
Demerara Rise	94	shale	1.10	0.02	0.240	1
Demerara Rise	94	shale	1.26	0.04	0.240	1
Demerara Rise	94	shale	1.27	0.05	0.250	1
Demerara Rise	94	shale	1.14	0.02	0.170	1
Demerara Rise	94	shale	1.22	0.03	0.250	1
Demerara Rise	94	shale	1.26	0.02	0.240	1
Demerara Rise	94	shale	1.20	0.02	0.200	1
Demerara Rise	94	shale	1.14	0.02	0.180	1
Demerara Rise	94	shale	1.08	0.02	0.240	1
Demerara Rise	94	shale	1.03	0.02	0.100	1
Demerara Rise	94	shale	1.13	0.02	0.280	1
Wynniatt	750	shale	1.03	0.04	0.040	1
Wynniatt	750	shale	1.12	0.05		1
Wynniatt	750	shale	1.44	0.02		1
Wynniatt	750	shale	0.66	0.04	0.030	1
Wynniatt	750	shale	1.53	0.05	0.030	1
Wynniatt	750	shale	-0.07	0.04	0.020	1
Wynniatt	750	shale	-0.05	0.04	0.030	1
Wynniatt	750	shale	1.97	0.04		1
Wynniatt	750	shale	1.80	0.05	0.040	1
Wynniatt	750	shale	1.11	0.04	0.020	1
Wynniatt	750	shale	1.79	0.02	0.020	1
Wynniatt	750	shale	1.20	0.03	0.030	1
Wynniatt	750	shale	0.30	0.04	0.020	1
Wynniatt	750	shale	0.64	0.03	0.020	1
Wynniatt	750	shale	1.52	0.03	0.020	1
Wynniatt	750	shale	1.31	0.03	0.030	1
Wynniatt	750	shale	1.29	0.06		1

Wynniatt	750	shale	0.81	0.03	0.030	[1]
Wynniatt	750	shale	0.70	0.06	0.030	[1]
Wynniatt	750	shale	1.60	0.03	0.020	[1]
Wynniatt	780	shale	0.07	0.03		[1]
Wynniatt	780	shale	-0.14	0.03		[1]

Supplementary References

1 Planavsky, N. J. *et al.* Low Mid-Proterozoic atmospheric oxygen levels and the delayed rise of animals. *Science* **346**, 635-638 (2014).

2 Bau, M. & Dulski, P. Distribution of yttrium and rare-earth elements in the Penge and Kuruman iron-formations, Transvaal Supergroup, South Africa. *Precambrian Res.* **79**, 37-55 (1996).

3 Gilleaudeau, G. J. *et al.* Oxygenation of the mid-Proterozoic atmospheric: clues from chromium isotopes and carbonates. *Geochemical Perspectives Letters* **2**, 178-187 (2016).

4 Cole, D. B. *et al.* A shale-hosted Cr isotope record of low atmospheric oxygen during the Proterozoic. *Geology*, doi:10.1130/G37787.1 (2016).

5 Gueguen, B. *et al.* The chromium isotope composition of reducing and oxic marine sediments. *Geochemica et Cosmochimica Acta* **184**, 1-19 (2016).

6 Reinhard, C. T. *et al.* The isotopic composition of authigenic chromium in anoxic marine sediments: A case study from the Cariaco Basin. *Earth Planet. Sci. Lett.* **407**, 9-18 (2014).

7 Frei, R., Gaucher, C., Stolper, D. & Canfie´ld, D. E. Fluctuations in late Neoproterozoic atmospheric oxidation - Cr isotope chemostratigraphy and iron speciation of the late Ediacaran lower Arroyo del Soldado Group (Uruguay). *Gondwana Research* **23**, 797-811, doi:10.1016/j.gr.2012.06.004 (2013).

8 Frei, R., Gaucher, C., Poulton, S. W. & Canfield, D. E. Fluctuations in Precambrian atmospheric oxygenation recorded by chromium isotopes. *Nature* **461**, 250-253, doi:10.1038/nature08266 (2009).
